# Sustained Physiological Stretch Induces Abdominal Skin Growth in Pregnancy

**DOI:** 10.1007/s10439-024-03472-6

**Published:** 2024-02-29

**Authors:** David Sachs, Raphael Jakob, Bettina Thumm, Michael Bajka, Alexander E. Ehret, Edoardo Mazza

**Affiliations:** 1 Institute for Mechanical Systems, ETH Zürich, Zurich, Switzerland; 2 Department of Obstetrics and Gynecology, University Hospital of Zurich, Zurich, Switzerland; 3 Swiss Federal Laboratories for Materials Science and Technology, Dubendorf, Switzerland

**Keywords:** Skin, Pregnancy, Mechanobiology, Growth, Mechanome, Multiphasic modeling

## Abstract

Supraphysiological stretches are exploited in skin expanders to induce tissue growth for autologous implants. As pregnancy is associated with large levels of sustained stretch, we investigated whether skin growth occurs in pregnancy. Therefore, we combined a mechanical model of skin and the observations from suction experiments on several body locations of five pregnant women at different gestational ages. The measurements show a continuous increase in stiffness, with the largest change observed during the last trimester. A comparison with numerical simulations indicates that the measured increase in skin stiffness is far below the level expected for the corresponding deformation of abdominal skin. A new set of simulations accounting for growth could rationalize all observations. The predicted amount of tissue growth corresponds to approximately 40% area increase before delivery. The results of the simulations also offered the opportunity to investigate the biophysical cues present in abdominal skin along gestation and to compare them with those arising in skin expanders. Alterations of the skin mechanome were quantified, including tissue stiffness, hydrostatic and osmotic pressure of the interstitial fluid, its flow velocity and electrical potential. The comparison between pregnancy and skin expansion highlights similarities as well as differences possibly influencing growth and remodeling.

**Supplementary Information:**

The online version contains supplementary material available at 10.1007/s10439-024-03472-6.

## Introduction

Mechanical forces shape biological tissues. The importance of forces in the development of life from the very beginning is increasingly appreciated [1]. Pressure, for example, induces jamming transitions in cell clusters from solid to fluid like states in the development of vertebrates [2]. Furthermore, forces generated through the heartbeat significantly influence heart morphogenesis [3]. Cells constantly feel and adapt to mechanical forces and deformation of the extracellular matrix (ECM). Stretches observed at the tissue length scale alter the "mechanical homeostasis" at cell length scale and induce cell responses [4]. Recent studies [5, 6] demonstrated that stretch-induced stiffening of the ECM in the proximity of a healing wound determines the level of scarring. The link between biomechanics and mechanobiology in the healing process of cutaneous wounds has been recently analyzed based on a multi-variable computational model by Pensalfini and Buganza Tepole [7]. In healthy skin stretching triggers keratinocyte proliferation and deposition of dermal matrix. This process is exploited in skin expanders to induce controlled skin growth for clinical applications [8, 9].

The link between tissue stretch and changes in the ECM is well documented (see e.g., [10]), yet the understanding of the associated mechanical and biological processes is still lacking. Investigating the interplay between tissue level stretch and alterations of the biophysical cell environment is part of what is called “mechanomics” [11]. Stretch induced alteration of the “mechanome” include variations of the geometry and stiffness of the solid extracellular matrix as well as changes in state variables of the interstitial fluid, such as its flow velocity and chemical potential. This stretch induced alterations depend on the local features of the ECM and are therefore tissue specific. For skin two main regions can be distinguished, the epidermis and the dermis. The epidermis consists of a multilayer configuration of confluent keratinocytes, so that global skin stretch directly translates into a corresponding level of cell stretch. Aragona et al. [9] demonstrated that stretch induced epidermal cell proliferation during skin expansion is associated with upregulation of the MEK-ERK pathway. Further they showed that in a subset of cells the YAP and MAL pathways were activated by the applied stretch. However, mechanotransduction in the two dermal layers, the papillary and the reticular dermis, is more complex due to the heterogenous matrix composition at cell length scale. Skin stretch induces local rearrangements of the collagen network, interstitial fluid displacement, and densification of the proteoglycan ground matrix [12, 13]. Resident cells are therefore exposed to a wide range of physical stimuli. These stimuli include changes in the chemical potential of the interstitial fluid, the shear stresses associated with its flow, the locally perceived stiffness of the collagen network, as well as its local strain state, which might differ from the tissue-level deformation of skin. So far, models proposed to rationalize observations of skin expansion [14–16] link the generation of new tissue with the level of applied homogeneous skin stretch. They thus neglect the various components of the dermal mechanome activated during skin expansion. On the other hand, some single and multiphase continuum and discrete mechanical models have been proposed and helped to understand growth and remodeling of tumors, blood vessels, and bones [17–20]. These models demonstrate that individual parts of the mechanome can drive growth, varying in each case. For instance, tumor growth was linked with matrix stress level [21] and endothelial cells proliferation was associated with flow induced shear stress [21–23].

The purpose of the present work is twofold: First, we asked the question if and to what extent skin growth occurs during pregnancy as a consequence of the sustained supraphysiological stretch of abdominal skin. To this end, we measured the response of abdominal skin to suction experiments at different time points along gestation and after delivery in five pregnant women. Based on a biphasic multilayer model of skin we showed that skin growth must be considered to rationalize the measurements. As a second step, we considered the various components of the skin mechanome and investigated if the biophysical cues activated during pregnancy differ from those in skin expansion. The paper is structured in the following way: We first present the experimental investigations of the skin’s response to suction along gestation. We use the computational model to analyze all measurements and determine the amount of skin growth needed to rationalize the experimental data. Utilizing the field variables calculated with the model of pregnancy, we analyze the changes of the mechanome during gestation. Based on existing literature, we then introduce a model of skin expansion, and compare the mechanome with the one of pregnancy. The results improve the understanding of tissue growth in human skin and may support future investigations on the pathophysiology of skin damage during pregnancy (skin marks) as well as on conditions associated with increased skin surface, such as lymphedema and obesity.

## Methods

### A Biphasic Nonlinear Model of Skin Growth and Resorption

As the mechanome includes contributions of the state variables characterizing the interstitial fluid, a poroelastic (finite strain biphasic) representation of the tissue was considered in the present work. Simulations are based on a biphasic multilayered skin model [12] which distinguishes the contributions of epidermis, papillary and reticular dermis, and adipose tissue which was amended to account for skin growth. Following existing growth models [24–27] the total deformation of the solid is decomposed into a passive mechanical contribution and a growth contribution. Accordingly, a multiplicative split to the deformation gradient ${\varvec{F}}$ is applied, yielding the mechanical part ${{\varvec{F}}}_{{\text{pm}}}$ and the growth tensor ${{\varvec{F}}}_{{\text{g}}}$:

1 $${\varvec{F}}={{\varvec{F}}}_{{\text{pm}}}{{\varvec{F}}}_{{\text{g}}}.$$

The strain energy function of the passive mechanical solid ${\varvec{\uppsi}}$ is modeled using a nonlinear and dissipative Rubin-Bodner type model [12, 28]

2 $${\varvec{\uppsi}}={\varphi }_{{\text{s}}}^{{\text{ref}}}\frac{{\mu }_{0}}{2q}\left[{\text{exp}}(qg)-1\right],$$

where ${\varphi }_{{\text{s}}}^{{\text{ref}}}$ corresponds to the solid volume fraction, and ${\mu }_{0}$ and $q$ are material parameters that vary for each skin layer. The function $g$ can be split into one matrix contribution (${g}_{{\text{m}}})$ and two fiber contributions, an elastic part (${g}_{{\text{fe}}}$) and a dissipative part (${g}_{{\text{fd}}}$), so that $g={g}_{{\text{m}}}+{g}_{{\text{fe}}}+{g}_{{\text{fd}}}$. The contributions are defined in the following way:

3 $${g}_{{\text{m}}}={m}_{1}\left({\text{tr}}\left({{\varvec{F}}}_{{\text{pm}}}{{\varvec{F}}}_{{\text{pm}}}^{T}\right)-3\right)+\frac{{m}_{1}}{{m}_{2}}\left[{J}_{{\text{pm}}}^{-2{m}_{2}}-1\right],$$

4 $${g}_{{\text{fe}}}=\frac{{m}_{3{\text{e}}}}{{m}_{4{\text{e}}}}\frac{1}{N}\sum_{i=1}^{N}{\langle {\lambda }_{{\text{fe}}}^{i}-1\rangle }^{2{m}_{4{\text{e}}}},$$

5 $${g}_{{\text{fd}}}=\frac{{m}_{3{\text{d}}}}{{m}_{4{\text{d}}}}\frac{1}{N}\sum_{i=1}^{N}{\langle {\lambda }_{f{\text{d}}}^{i}-1\rangle }^{2{m}_{4{\text{d}}}}.$$

Therein, ${m}_{1},$ ${m}_{2}$, ${m}_{3{\text{e}}}$, ${m}_{4{\text{e}}}$, ${m}_{3{\text{d}},}$ and ${m}_{4{\text{d}}}$ are material parameters and $N$ corresponds to the number of fiber families with referential directions ${{\varvec{R}}}_{i}$($\Vert {{\varvec{R}}}_{i}\Vert =1$) following an equiangular distribution in the plane with a slight out-of-plane pitch as described in Wahlsten et al. [13]. The Macaulay brackets $\langle \cdot \rangle$ indicate fibers being only active in tension. The stretches in the direction of the fibers are denoted with ${\lambda }_{{\text{fe}}}$ for the elastic and ${\lambda }_{{\text{fd}}}$ for the dissipative contribution. Finally, ${J}_{{\text{pm}}}$ denotes the determinant of the mechanical part of the deformation gradient: ${J}_{{\text{pm}}} =\mathrm{ det}({{\varvec{F}}}_{{\text{pm}}})$.

Evolution equations for the dissipative fibers' contributions are implemented as in Wahlsten et al. [13, 29] and Sachs et al. [12]

The motion of the fluid is governed by the fluid chemical potential ${\mu }_{{\text{f}}}$ [30]

6 $${\mu }_{{\text{f}}}=\frac{p}{{\rho }_{{\text{f}}}}-\frac{\Delta \pi }{{\rho }_{{\text{f}}}}+{\mu }_{{\text{f}},0}.$$

Therein $p$ is the hydrostatic and $\Delta \pi$ the osmotic pressure, ${\rho }_{{\text{f}}}$ is the density of the fluid, which is assumed to be constant, and ${\mu }_{{\text{f}},0}$ is the reference chemical potential. The interstitial fluid velocity ${{\varvec{j}}}_{{\text{f}}}$ is related to the fluid chemical potential through Darcy’s law

7 $${{\varvec{j}}}_{{\text{f}}}=-{\varvec{k}}{\rho }_{{\text{f}}}\mathrm{ grad}\left({\mu }_{{\text{f}}}\right).$$

In Eq. (7), ${\varvec{k}}$ describes the spatially isotropic permeability tensor, which was chosen with a simple deformation dependent form [12]. The biphasic theory links the solid phase kinematics and the fluid flow based on the assumption of incompressibility of each phase. The conservation of mass and momentum are given by [27, 31]:

8 $${\text{div}}\left({{\varvec{v}}}_{{\text{s}}}+{{\varvec{j}}}_{{\text{f}}}\right)={\dot{V}}_{{\text{g}}},$$

9 $${\text{div}}\left({\varvec{\sigma}}\right)=0,$$

where ${\dot{V}}_{{\text{g}}}$ accounts for the rate of volume change due to growth, ${{{\varvec{v}}}_{\text{s}}}$ denotes the spatial velocity field of the solid phase and ${\varvec{\sigma}}$ is the Cauchy stress tensor of the mixture resulting from the summation of the solid stress ${{\varvec{\sigma}}}_{{\text{s}}}$ and the hydrostatic pressure $p$ as : ${\varvec{\sigma}}= {{\varvec{\sigma}}}_{{\text{s}}}+p {\varvec{I}}$ [32].

Glycosaminoglycans and proteoglycans provide the matrix of skin with fixed negative charges. The fixed charge density depends on the passive mechanical deformation and governs the cations and anions concentrations ($${c}_{+},{c}_{-})$$ of the interstitial fluid. The difference of concentrations in the fluid induces a deformation dependent electrical potential $$\Psi$$ [33]

10 $$\Psi \left({J}_{{\text{pm}}}\right)=\frac{RT}{2F}{\text{ln}}\left(\frac{{{\text{c}}}_{-}\left({J}_{{\text{pm}}}\right)}{{c}_{+}({J}_{{\text{pm}}})}\right).$$

Therein $R$ is the universal gas constant, $T$ the temperature, and $F$ Faraday’s constant. Based on previous formulations we model anisotropic in-plane skin growth as a strain driven phenomenon [14, 15, 34]. The growth deformation tensor is thus given by

11 $${{\varvec{F}}}_{{\text{g}}}= {\lambda }_{{\text{g}},1}{{\varvec{G}}}_{1}\otimes {{\varvec{G}}}_{1}+{\lambda }_{{\text{g}},2}{{\varvec{G}}}_{2}\otimes {{\varvec{G}}}_{2}+{\varvec{N}}\otimes {\varvec{N}}$$

With ${{\varvec{G}}}_{1}$ and ${{\varvec{G}}}_{2}$ corresponding to the cranial-caudal and lateral direction, respectively, and ${\varvec{N}}$ to the plane normal. This assumption is related to the fact that in pregnancy, as in skin expansion, the tissue experiences in plane stretch and out of plane contraction. Each growth stretch ${\lambda }_{{\text{g}},i}$ in equation (11) is then calculated by the following constitutive evolution equation [35]

12 $${\dot{\lambda }}_{{\text{g}},i}=k{\langle {\lambda }_{{\text{pm}},i}-{\lambda }^{{\text{G}}}\rangle }^{b}-k{ \langle {\lambda }^{{\text{R}}}-{\lambda }_{{\text{pm}},i}\rangle }^{b}.$$

The first term of the evolution equation describes tissue growth and the second term tissue resorption. The variable ${\lambda }_{{\text{pm}},i}=\Vert {{\varvec{F}}}_{{\text{pm}}} {{\varvec{R}}}_{i}\Vert$ thereby describes the current passive mechanical strain in the corresponding direction. ${\lambda }^{{\text{G}}}$ and ${\lambda }^{{\text{R}}}$ denote the critical strain values, above and below which growth and resorption occur, respectively. The parameter $k$ describes a rate constant, which was set equal for growth and resorption in this model and the exponent $b$ is a material parameter governing the nonlinearity of the growth and resorption law. The nonlinearity parameter $b$ and the rate factor $k$ were adjusted such that the evolution of growth stretches for skin expansion reported in [15, 16] were qualitatively reproduced in terms of shape and order of magnitude. The thus obtained values of $b=2$ and $k = 0.0001\left[\frac{1}{{\text{s}}}\right]$ were thus used in all simulation of both skin expansion and pregnancy.

### The Passive Mechanical Behavior of Human Skin

The parameters selected to describe the passive mechanical behavior of each skin layer are based on our previous work [12, 13]. Using data from ex vivo uniaxial and in vivo biaxial suction experiments the parameters of the biphasic multilayer model were determined through inverse analysis, as described in Supporting Information section "Introduction".

**Fig. 1:**
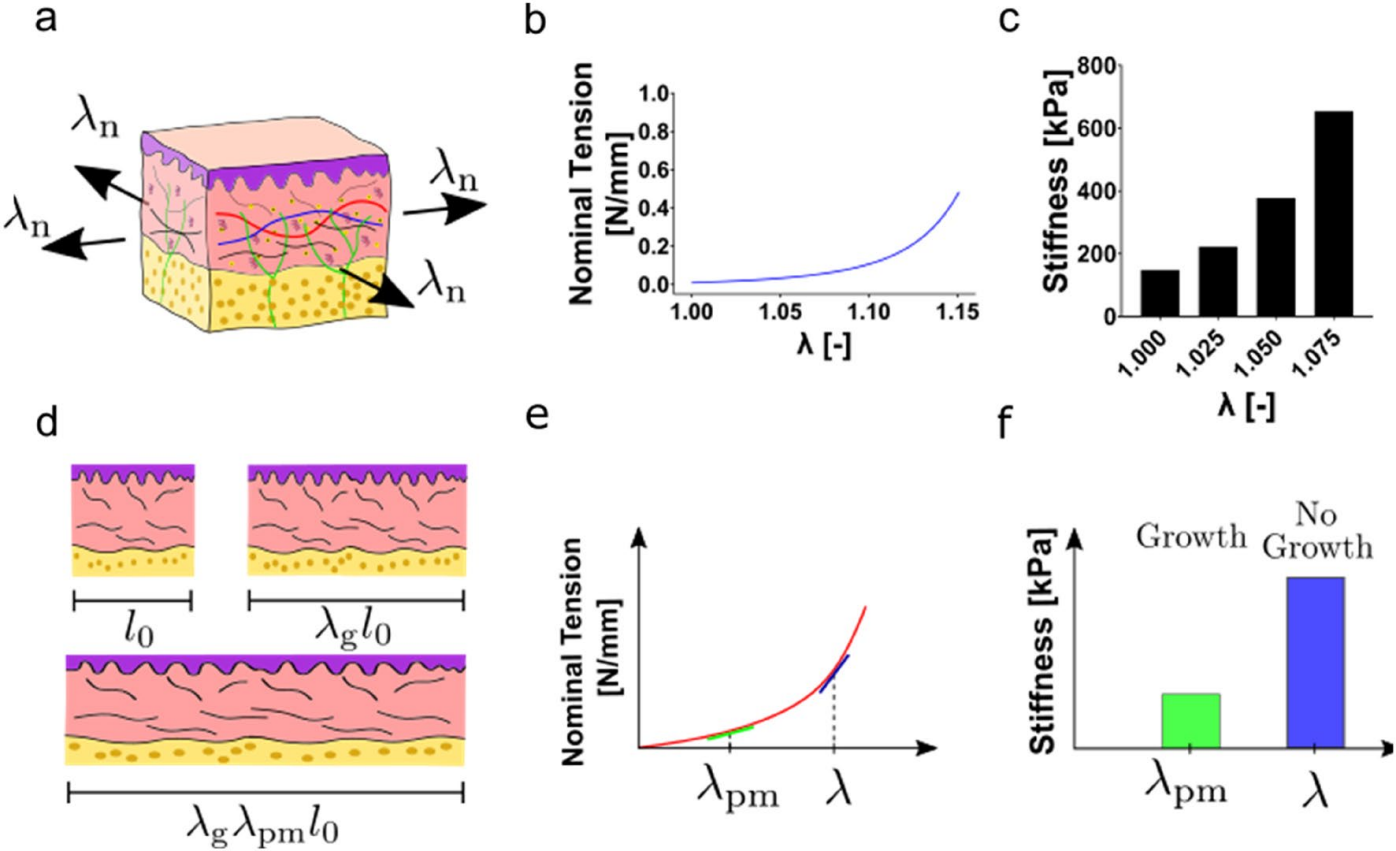
**a** The prestretch ${\lambda }_{\text{n}}$ can be used to represent increasing skin stretch associated with pregnancy. **b** the monotonic equibiaxial response of skin shows a typical J-shaped stress-strain relationship. **c** The stiffness of skin increases nonlinearly with the applied prestretch ${{\lambda }_{\text{n}}}$. **d** Starting from an initial length ${l}_{0}$ tissue grows to a length of ${\lambda }_{\text{g}}{l}_{0}$ and then is further stretched to ${\lambda }_{\text{g}}{\lambda }_{\text{pm}}{l}_{0}$. **e** Growth thus reduces the passive mechanical stretch ${\lambda }_{\text{pm}}$ and the nominal tension as well as the **f** tangent stiffness and thereby softens the current passive mechanical response indirectly

To adapt the model to different in vivo tension states, as occurring during pregnancy, the in vivo pre-stretch ${\lambda }_{{\text{n}}}$ is applied equibiaxially in the skin plane, as shown in Fig. 1a. Due to the non-linearity of the biaxial tension-stretch relationship (Fig. 1b), larger pre-stretch leads to increasing in-plane tangent stiffness of skin (Fig. 1c). Thus, increasing ${\lambda }_{{\text{n}}}$ allows to adapt the model to stiffer skin conditions associated with increased skin surface during pregnancy. On the other hand, tissue growth reduces the magnitude of the passive mechanical stretch and therefore of the elastic deformation associated with ${\lambda }_{{\text{n}}}$.Neglecting inelastic passive mechanical deformation, the total stretch $\lambda$ can be decomposed into a growth stretch ${\lambda }_{{\text{g}}}$ and an elastic passive mechanical stretch ${\lambda }_{{\text{pm}}}$ (Fig. 1d). For a given total stretch ${\lambda }_{{\text{n}}}$, the bigger the growth stretch, the smaller the passive mechanical stretch, so that the current stiffness of the tissue is reduced. If both the total stretch of the tissue and its current stiffness are known, computational analysis offers the possibility to infer the passive mechanical and the growth stretch of the tissue in the current state.

### Suction Experiments

**Fig. 2:**
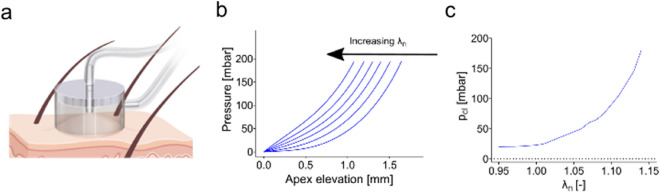
**a** Drawing of the suction device NIMBLE **b** the computationally obtained elevation-pressure curves confirm that maximum apex displacement decreases with increasing elastic stretch **c** the closing pressures for an elevation of d = 0.5 mm show a high sensitivity of the closing pressure to changes in equibiaxial stretch

In vivo suction measurements are often utilized to determine the resistance of skin to deformation. In the present study, the suction device “NIMBLE” [36] is employed for all the suction measurements (Fig. 2a). As previously described [36], when conducting a measurement a circular suction chamber is placed on the skin surface. Subsequently, varying negative pressure (suction) is applied to the confined surface through a narrow tube within the suction chamber. The narrow tube is separated from the skin by a preset distance. Suction is then incrementally elevated until the skin displacement reaches the preset original gap between skin and suction tube, thereby closing the tube opening. The amount of negative pressure gap (p_cl_: called “closing pressure”) needed to close the gap then serves as indication for skin stiffness. The present measurements were performed using a suction chamber opening diameter of 6 mm and a maximum tissue elevation of 0.5 mm.

To illustrate the influence of the mechanical in vivo prestretch ${\lambda }_{{\text{n}}}$ on the suction response, a parametric study was performed in which the experiment up to a maximum suction pressure of 200 kPa was simulated for different stretches ${\lambda }_{{\text{n}}}$ ranging from 0.95 to 1.15. As shown in Fig. 2b an increase in ${\lambda }_{{\text{n}}}$ results in a stiffer mechanical behavior of the skin, which reduces the maximum apex displacement reached for the specified pressure. Correspondingly, the closing pressure for a height of 0.5 mm directly correlates with the level of applied ${\lambda }_{{\text{n}}}$ as shown in Fig. 2(c). For small strains the relationship of closing pressure and applied prestrain is almost linear, when increasing the strain, it however becomes nonlinear.

**Fig. 3:**
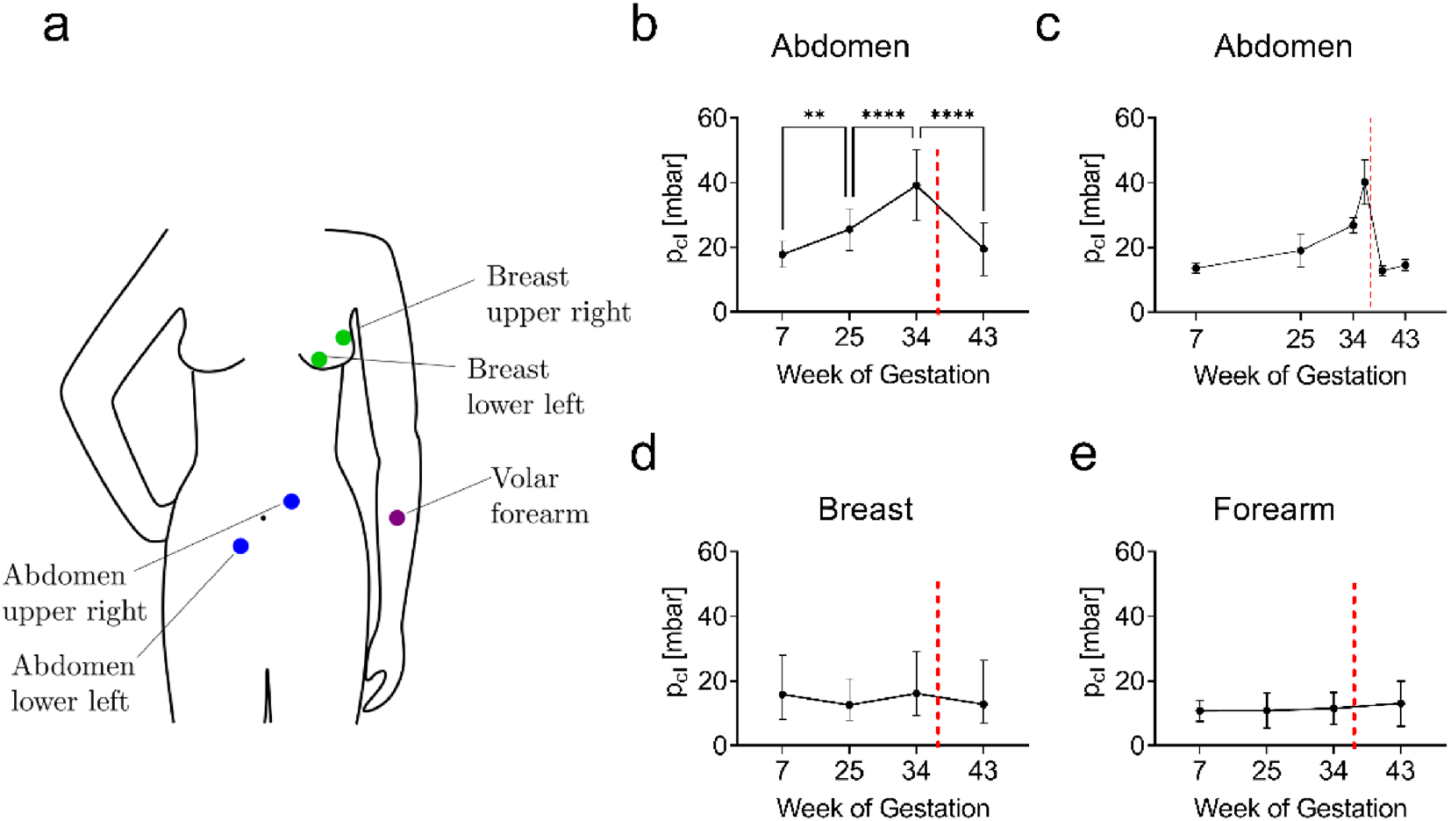
**a** Suction measurements on 5 pregnant women were performed on the forearm and two locations of both abdomen and breast. **b** Closing pressures on the abdomen show a significant increase during pregnancy, with a reduction to normal levels post-partum. **c** Additional measurements on the abdomen of one individual show a strong increase in stiffness immediately before birth and an abrupt decrease after birth. **d** Breast and **e** volar forearm show no significant changes during pregnancy. Level of significance: *p > 0.05, **p > 0.01, ****p > 0.0001. Error bars indicate standard deviation. The red line indicates the predicted time point of delivery

Five pregnant women were included in the study (approval of the local ethics committee, EK 2020-N-174). Experiments were carried out at five different locations, i.e., the upper right and lower left abdomen, the upper right and lower left breast as well as the volar forearm, as can be seen in Fig. 3. Measurements were conducted at three timepoints before birth, i.e., gestational weeks 7, 25 and 34, as well as 6 weeks post-delivery. For each location, a triplicate of measurements was performed at each timepoint by one single operator. After verification of their equivalence, the measurements of the two locations of the abdomen and of the two locations of the breast were averaged. For one participant additional measurements were performed 2 weeks before and after birth.

### Statistical Analysis

The statistical analysis was performed using GraphPad Prism (GraphPad Software Inc., San Diego, USA). An ordinary one-way ANOVA test was performed, followed up by the Holm-Sidak post-hoc test. The level of significance was p < 0.05.

## Results

### Skin Stiffening and Growth During Pregnancy

In this section we first present the results of the experiments on pregnant women. We then apply the model to rationalize the progression of suction resistance.

#### Measuring the Passive Mechanical Response of Skin During Pregnancy

The results of the experimental study are summarized in Fig. 3. The abdomen (Fig. 3b), shows a significant difference in the closing pressure at the different time points during pregnancy. Higher pressure correlates with stiffer tissue response. The pressure increased from a mean value of 20 mbar at week 7 to 25 mbar at week 25 to 40 mbar at week 34. The measured values decrease after birth, down to a level that does not differ significantly from the value at the beginning of pregnancy. For one individual, additional measurements were performed two weeks before and two weeks after birth, (Fig. 3c). The data show a strong increase in stiffness in the time immediately before birth, with closing pressures reaching about four times the initial level. Further, the closing pressure strongly drops two weeks after pregnancy and recovers by a few mbar between two weeks and six weeks after birth. For both breast and volar forearm no significant trends are observed throughout pregnancy (Fig. 3d, e). This indicates that systemic changes associated with the hormonal status do not affect the stiffness of the skin to a measurable extent.

#### Skin Growth Is Required to Rationalize the Passive Mechanical Response during pregnancy

**Fig. 4:**
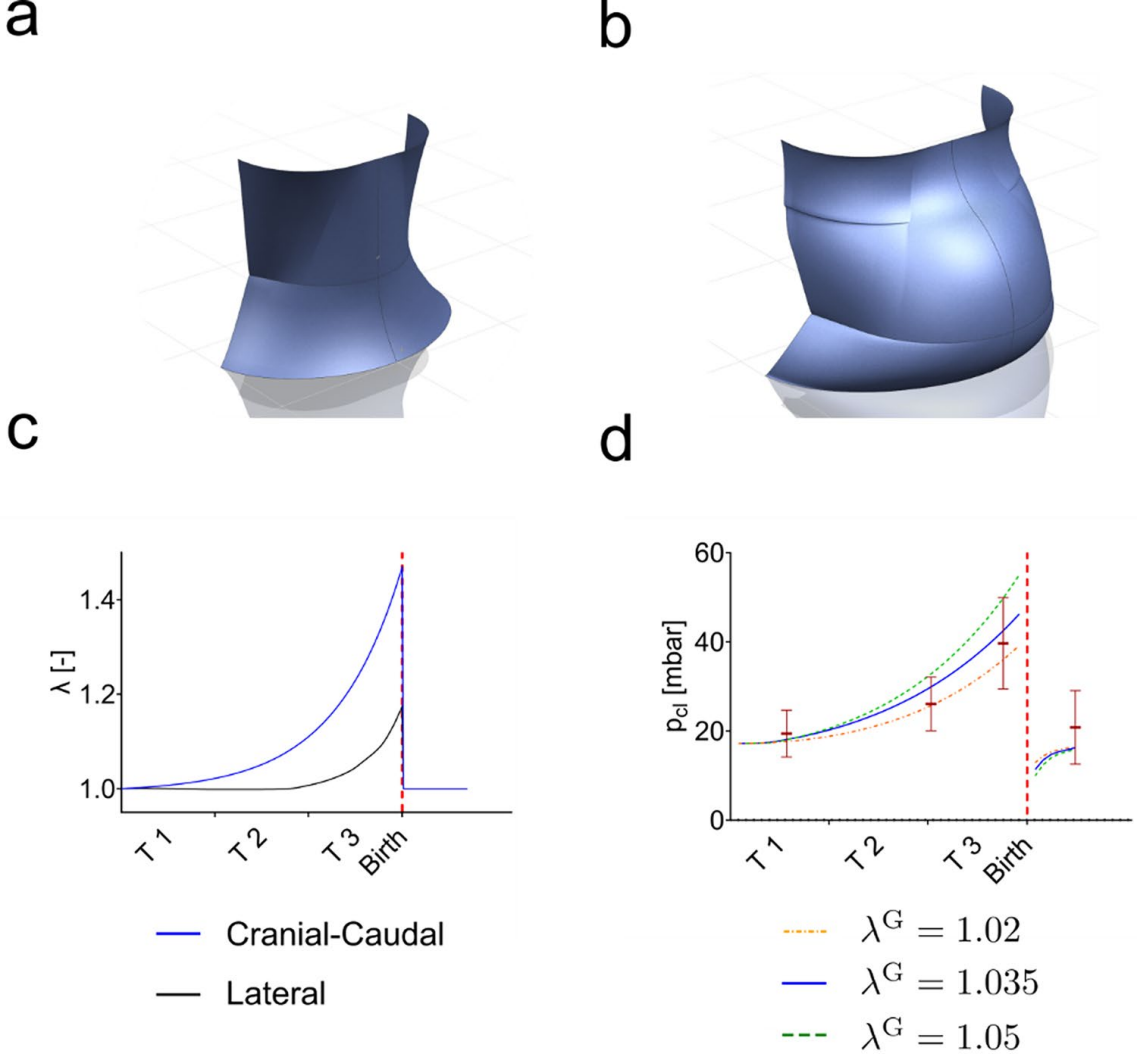
Model of the abdomen created to calculate cranial-caudal and lateral strain during pregnancy for **a** 8 weeks and **b** 37 weeks. **c** Pregnancy-related deformation of skin is modeled by applying stretches in two in-plane directions, cranial-caudal and lateral. For both stretches the largest rates of increase occur in the last trimester. **d** Predicted closing pressure based on a model with different levels of ${\lambda }^{{\text{G}}}$

To estimate the total stretches occurring during pregnancy a three-dimensional model of the female abdomen was created in the CAD Software NX™ (Siemens AG, Munich, Germany). Models were informed from data available in the literature [37]. The models for two timepoints in gestation, 7 weeks and 37 weeks, respectively, are shown in Fig. 4a, b. Based on the changes in abdominal dimensions, total stretches in cranial-caudal and lateral direction were quantified. Note that this approach provides only average stretches over the whole abdomen and no information is available on the local deformation in each abdominal region. As indicated in Fig. 4c, both stretches show the largest rate of increase in the last trimester (T 3). The maximum stretch reaches 1.4 in cranial-caudal direction and 1.18 in lateral direction. These stretch values were imposed as total in-plane skin deformations in a simulation of the corresponding suction response of abdominal skin. Model parameters used to represent the mechanical behavior of skin are listed in Table S1. They were determined based on an inverse analysis of uniaxial and biaxial experiments performed in vivo and ex vivo on human skin [12], see Supplementary section "Introduction". Even considering the variability of model parameters for skin at different body locations or from different subjects, the computationally obtained closing pressures exceed the experimentally measured ones by an order of magnitude. The experimental data can only be rationalized when growth is activated in the model. Thereby, the most influential parameter is the critical growth stretch ${\lambda }^{{\text{G}}}$. Results of the predicted closing pressures are shown in Fig. 2d for three different critical growth stretches (${\lambda }^{{\text{G}}}$=1.02, 1.035, 1.05), with larger growth for lower values of ${\lambda }^{{\text{G}}}$_._ The critical stretch for resorption was set to ${\lambda }^{{\text{R}}}= 0.98$ for all three cases. It should be noted that growth and resorption stretches are reported with respect to the in vivo reference configuration. Skin is usually not stress free in its in vivo reference configuration as resulting from an initial pre-swelling to equilibrate the osmotic pressure due to the presence of fixed charges in the tissue [13] as well as a superimposed biaxial tension to represent its physiological tensional state [12]. For this reason, a resorption stretch ${\uplambda }^{{\text{R}}}<1$ does not necessarily imply compression. As stretch is applied during pregnancy the closing pressure increases, depending on the critical stretch ${\lambda }^{{\text{G}}}$. In the initial phase ${\lambda }^{{\text{G}}}=1.02$ seems to better reproduce the experimental data. At the last time point, the simulation of ${\lambda }^{{\text{G}}}=1.035$ shows the best correlation with the average closing pressure, while the other two fall within the upper and lower range of the standard deviation of the measurements. After pregnancy the closing pressure drops as the stretch is released. Interestingly, the closing pressures fall below the initial values. To recover the initial closing pressure, the in-plane passive mechanical strains in skin need to increase, which is achieved by resorption of tissue.

### Changes of the Skin Mechanome During Pregnancy

**Fig. 5:**
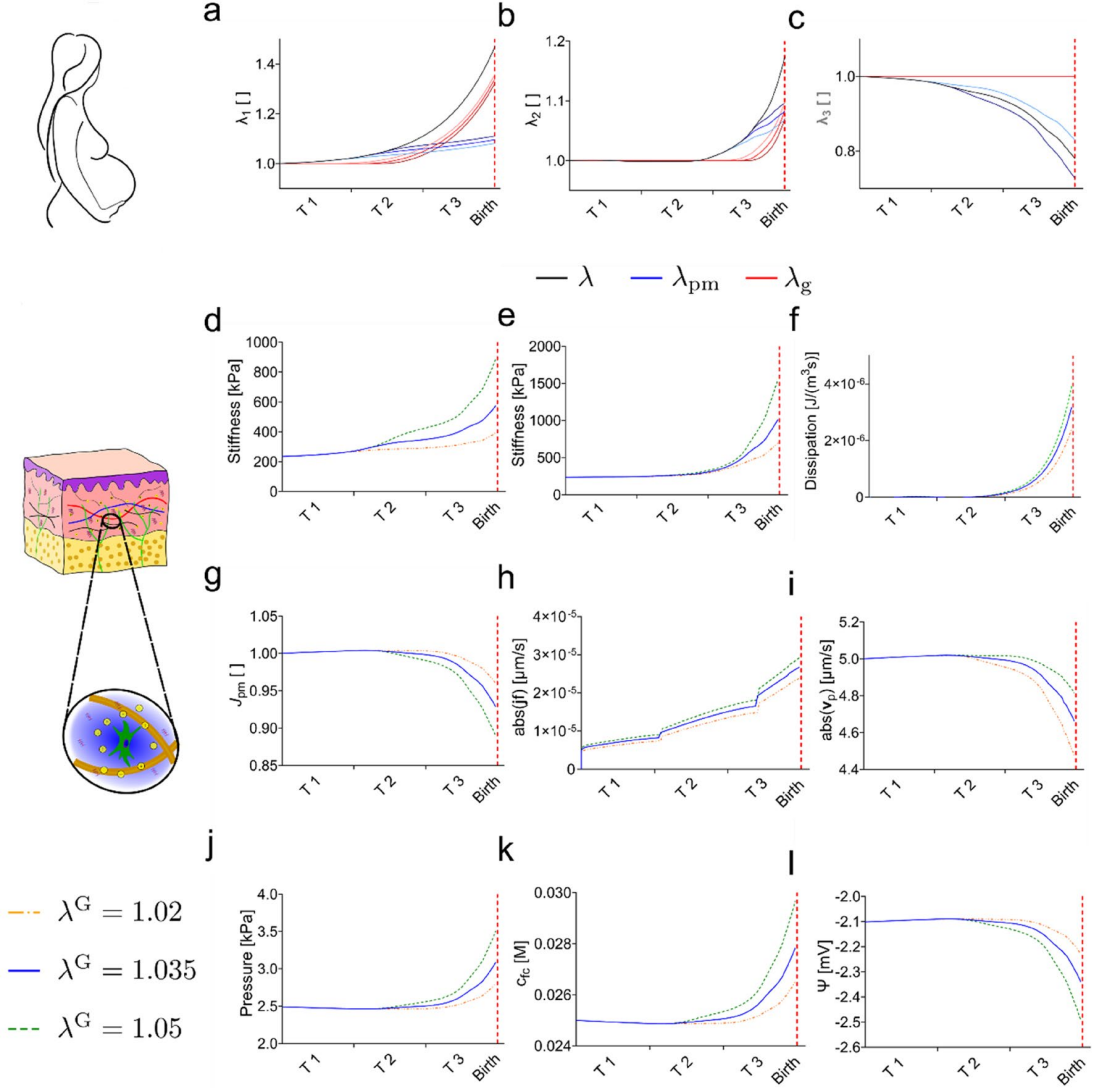
Simulations using three levels of critical stretch ${\lambda }^{{\text{G}}}$ indicate tissue growth and changes in various components of the mechanome are larger in the last trimester of pregnancy. Passive mechanical stretches (blue) and growth stretches (red) are shown for **a** cranial-caudal direction, **b** lateral direction, **c** out-of-plane direction. The various components of the mechanome are shown: **d** stiffness in the cranial-caudal direction, **e** stiffness in lateral direction, **f** energy dissipation in the solid material, **g** volume ratio, **h** interstitial fluid velocity, **i** perfusion velocity in skin, **j** osmotic and hydrostatic pressure, **k** fixed charge density, **l** electrical potential. The red line indicates the expected time point of delivery.

The model allows determining all internal field variables associated with the various components of the mechanome, varying as a consequence of stretch during pregnancy. The results of the simulation are shown in Fig. 5 for the reticular dermis. The passive mechanical stretches and the growth stretches for the three cases of ${\lambda }^{{\text{G}}}$ are shown in Fig. 5a–c. During the first two trimesters the in-plane deformations remain small, and no significant growth occurs in either direction. In the last trimester stretches increase rapidly and exceed the critical thresholds so that growth occurs. At birth the model predicts a passive mechanical stretch of 1.1 in both directions and growth stretches of around 1.3 in the cranial caudal direction and 1.1 in the lateral direction, respectively. In out of plane direction, depicted in Fig. 5c, the tissue contracts due to the in-plane passive mechanical stretches. The out-of-plane total stretch is not prescribed but governed by the mechanical behavior of the skin.

From the state of deformation all variables of the mechanome can be determined, as explained in the Supporting Information Sect. "Results". As expected, the components of the mechanome follow the same trend as for the passive mechanical stretch (Fig. 5d–l). During the first two trimesters of pregnancy only little change is observed. The main deviation from the initial state occurs during the last trimester. The magnitude of deviation is larger for higher values of critical stretch, i.e., less growth. The stiffness of the tissue increases in both principal directions from about 200 kPa to about 500–1100 kPa for both the cranial caudal direction (d) and the lateral direction (e). Due to the slow rates of stretches during pregnancy, which are in the order of $\dot{\lambda }=0.1\lceil\frac{1}{{\text{month}}}\rceil$, the dissipation rate of the solid is negligible and several orders of magnitude smaller than the rate of change of the strain energy density (f). The interstitial fluid velocity associated with efflux of fluid due to volume changes is smaller than $0.001\frac{\mathrm{\mu m}}{\mathrm{s}}$ in pregnancy as shown in (h). Further, we calculated the perfusion velocity being the velocity of the flow of interstitial fluid that leaks from the capillaries into the interstitial space and is subsequently reabsorbed by the lymphatic capillaries. This fluid flow mainly depends on the permeability of the tissue and its calculation is described in Sect. "Results" of the Supporting Information. The perfusion velocity reduces due to a passive stretch induced decrease in permeability from $5\frac{\mathrm{\mu m}}{{\text{s}}}$ to the lowest value of $4.6\frac{\mathrm{\mu m}}{{\text{s}}}$ in the weeks before birth. Skin volume change, shown in (g), is in a moderate range with a reduction of volume before birth of 5 to 10% relative to its initial value. Hydrostatic and osmotic pressures are equal for slow deformation rates. The corresponding curve is plotted in (j) and show maximum changes of about 1 kPa. The fixed charge density (k) increases during pregnancy from its initial values of 0.025 M to 0.029 M. The electrical potential (l) reduces by about 0.3 mV from − 2.1 mV to − 2.4 mV.

### Comparison with the Mechanome of Skin Expansion

**Fig. 6:**
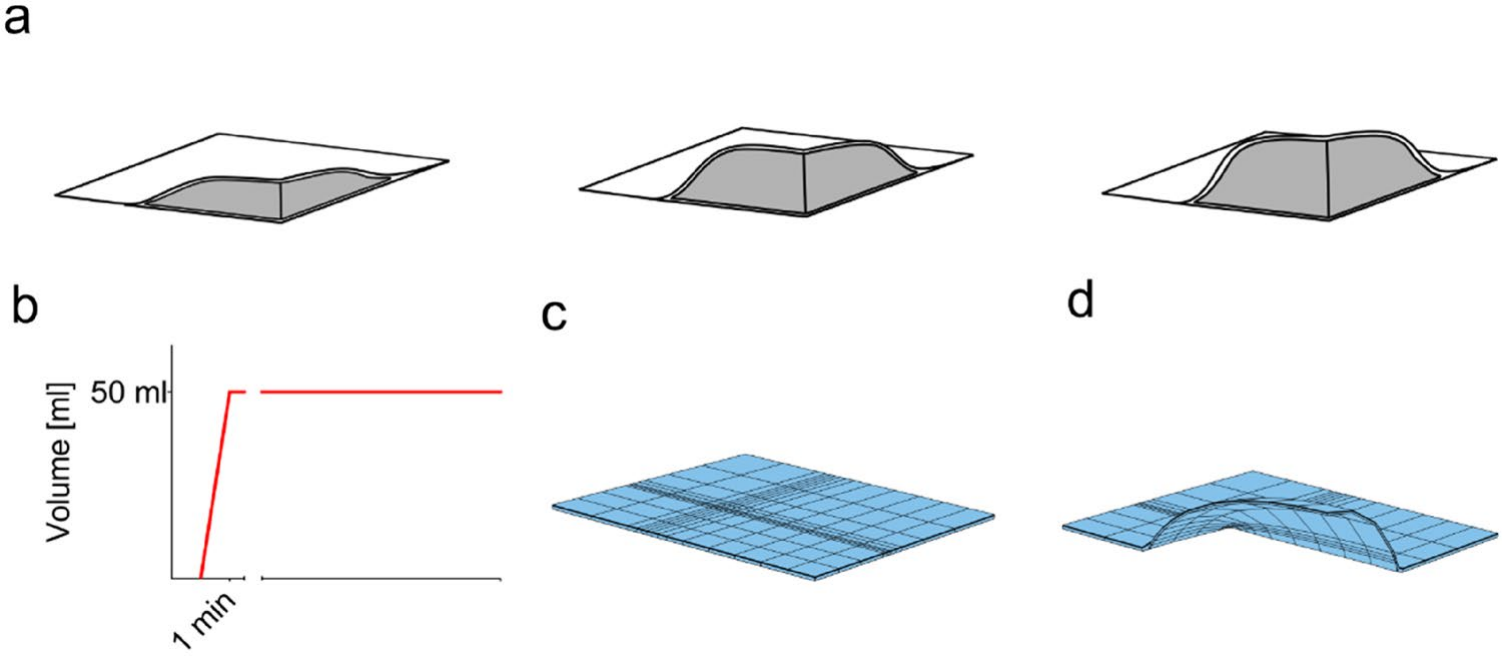
**a** During skin expansion an expander (gray) is implanted below the dermis and inflated in multiple steps until the desired area of new tissue is obtained, **b** the protocol in this study assumes an inflation of 50 ml within 1 minute, afterward the volume is held constant for 1 week before another cycle of inflation is started. The finite element mesh of the expander is shown in the **c** initial configuration and **d** final deformed configuration after the inflation protocol was applied four times.

The same biphasic skin model was used to simulate tissue growth during skin expansion. For the expansion process a fluid filled cavity is implanted below the reticular dermis [9, 16, 38]. The cavity is then gradually inflated as sketched in Fig. 6a. In our simulation, (Fig. 6b), the expander of dimensions $60 \times 100 {{\text{ mm}}}^{2}$ is inflated with 50 ml of fluid within 1 min. The inflation step is repeated four times with 1 week interval between each step. The finite element model of the expander is shown in the initial state (c) and the final state (d). For the growth calculation we used the same model parameters as for pregnancy and the same three values of critical stretch were considered in the simulations, (${\lambda }^{{\text{G}}} =1.02, \mathrm{1.035,1.05}$). Further details on the model implementation and the boundary conditions are reported in Sec. "Discussion" of the Supporting Information.

**Fig. 7:**
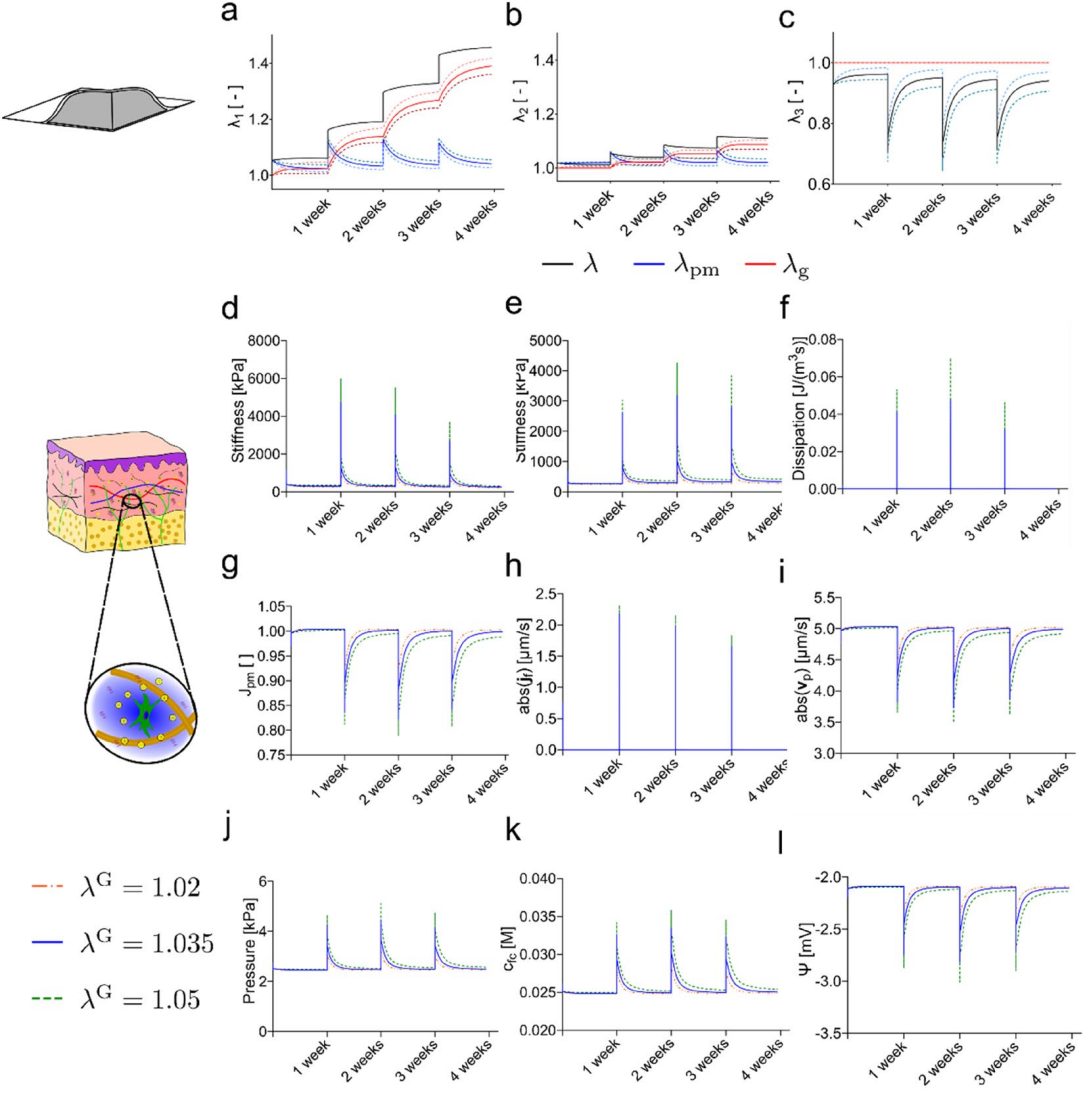
Skin growth is triggered by inflating the expander every week. Simulations were performed using three levels of critical stretch. Results indicate that tissue growth and changes in various components of the mechanome are larger in the phase immediately following expander inflation. Passive mechanical stretches (blue), growth stretches (red), and the total stretch (black) are shown for **a** cranial-caudal direction, **b** lateral direction, **c** out-of-plane direction. The various components of the mechanome are shown: **d** stiffness in the first principal direction, **e** stiffness in second principal direction, **f** energy dissipation in the solid material, **g** volume ratio, **h** interstitial fluid velocity, **i** perfusion velocity in skin, **j** osmotic and hydrostatic pressure, **k** fixed charge density, **l** electrical potential

During skin expansion skin grows in in-plane direction after each increase of expander volume (Fig. 7a, b). At each expansion step the skin is stretched by about 5 to 10% in direction parallel to the short edge of the expander and about 2 to 5% in direction parallel to the longer edge of the expander. While little growth occurs in the first week of expansion, skin grows significantly in both directions from the second week of expansion. Skin is almost fully grown to a point where the passive mechanical stretch almost reaches the critical growth stretch again just before the next expansion step. The rapid inflation during the expansion step leads to a strong out-of-plane contraction. From the second cycle onwards, skin contracts in out-of-plane direction by about 30% at the beginning of the expansion step, as shown in Fig. 7c. The level of skin growth obtained with the present model are well in line with the results of previous studies [9, 15, 16] with about 40% of increase in skin area attained after 4 weeks.

The changes in the various components of the mechanome during skin expansion resemble the temporal characteristics of the passive mechanical stretches. Each inflation step induces a sharp peak in each component of the mechanome, followed by a relaxation to almost the initial level. The results of the components of the mechanome in the reticular dermis are depicted in Fig. 7d–l. Stiffness increases in both principal directions (d, e) to few MPa with highest values of 6 MPa reached for the first principal direction and 4 MPa for the second principal direction. Similar temporal evolutions are observed for volume ratio (g), perfusion velocity (i), hydrostatic and osmotic pressure (j), fixed charge density (k), and electrical potential (l). Skin thereby exhibits a volume reduction of about 20% during the process of expansion. The perfusion velocity decreases due to decrease of permeability by 1 $\frac{\mathrm{\mu m}}{{\text{s}}}$ from 5 $\frac{\mathrm{\mu m}}{{\text{s}}}$ to 4 $\frac{\mathrm{\mu m}}{{\text{s}}}$. The fixed charge density increases due to the compaction from 0.025 to 0.35 M leading to a change in osmotic pressure from 2.5 kPa to about 5.5 kPa. The electrical potential decreases by almost 1 mV with a minimum value of about − 3 mV. During the inflation step the deformation rates are in the range of $\dot{\lambda }=0.1\left[\frac{1}{{\text{minute}}}\right]$. This deformation rate is fast enough to trigger time dependent effects which are incorporated in the biphasic nonlinear model. The rate of energy dissipation is up to 1% of the rate of change of strain energy density. Both the dissipation of the solid (f) and the fluid velocities (h) show distinct peaks at the inflation step. Maximum velocities to be expected are about 2 $\frac{\mathrm{\mu m}}{{\text{s}}}$. The changes of each variable in the transient phase immediately following the inflation step are reported in Sec. 5 of the Supporting Information.

## Discussion

### Combining Suction Measurements and Mechanical Simulations Allows Quantifying Skin Growth and Resorption

Suction measurements follow a similar trend for all subjects, with progressive increase of stiffness (larger negative pressure needed for the same skin elevation) along gestation. In line with changes in abdominal dimensions, largest values of stiffness are measured in the last trimester. Further, skin stiffness decreases below its initial values after delivery. Our experimental results are in line with previous results obtained by in vivo suction measurements using another commercially available device [39]. Therein, both phenomena, an increased stiffness before delivery and a decreased stiffness after delivery were observed, with some study participants showing decreased values even 4 months after delivery.

The constitutive equations applied to predict skin growth in pregnancy are based on a growth model developed to rationalize skin expansion. Importantly, it seems that the same formulation and a similar value of critical growth stretch provides a reasonable representation of tissue growth in skin expansion and in pregnancy. Moreover, the present model formulation also provides a quantification of all relevant components of the skin mechanome, indicating important differences in terms of cell level stimuli between short-term growth in tissue expansion and long-term growth in pregnancy, see section "The Components of the Mechanome and Their Changes with Skin Stretch".

Changes in skin stiffness are observed to be negligible for breast and forearm. We consider this finding as a confirmation that (i) no significant systemic alterations of skin take place along gestation and (ii) the passive mechanical skin model can be considered as representative for the whole pregnancy. Without growth, the model indicates that abdominal deformation would lead to much higher levels of closing pressure than the measured values. Thus, the results support the conclusion that skin growth occurs during pregnancy. This is in line with previous reports, which showcased the usability of excessive tissue after pregnancy for surgical applications [40–42]. Calculations indicate a critical stretch level between 1.02 and 1.05 to trigger skin growth, with corresponding levels of maximum skin surface increase of 37% to 43%. Importantly, growth occurs mainly in the third trimester of gestation.

Measurements of suction resistance after delivery and corresponding simulations point at the relevance of tissue resorption. The excess tissue generated during pregnancy creates a condition of skin slackness evident in the simulation results (Fig. 4) and in line with measurements two weeks after delivery (Fig. 3). At the later timepoint (6 weeks after delivery) the initial stiffness is regained, suggesting that the physiological level of skin tension is built up through tissue resorption within this time span.

### The Components of the Mechanome and Their Changes with Skin Stretch

**Fig. 8:**
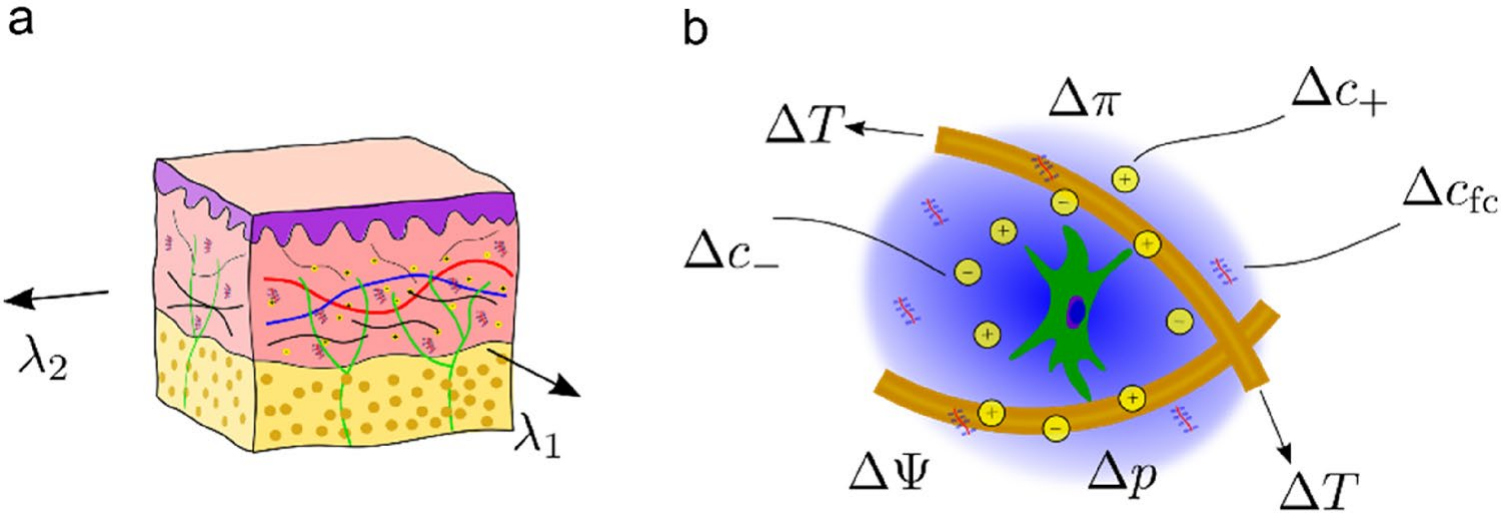
Dermal cells reside in a multiphasic environment, a global deformation (${\lambda }_{1},{\lambda }_{2})$ at the macroscale **a** results in a local change of various chemo-mechanical quantities at the length scale of cells (**b**), such as collagen fiber tension $\Delta T$, hydrostatic pressure $\Delta p$, osmotic pressure $\Delta \pi$, as well as fixed charge density $\Delta {c}_{\text{fc}}$, anion and cation concentrations ($\Delta {c}_{-},\Delta {c}_{+})$ and electrical potential $\Delta \Psi$

During pregnancy skin is stretched by the growing fetus over a duration of about 9 months resulting in various biological changes of skin [43]. Most common skin changes in pregnancy are hyperpigmentation, stretch marks and vascular spiders. While stretch marks are observed on the abdomen and the breast, the other changes are also observed on other areas of the body [44]. Similar as for skin expansion, sustained stretch modifies the morphology and properties of dermal ECM and thus the skin mechanome. In fact, cells sense a multitude of stimuli from their biophysical environment [45] which are altered in case of tissue deformation. The extracellular matrix of skin is composed of collagen and elastin fibers as well as the charged groups of the glycosaminoglycans. The void spaces of this charged matrix are filled with interstitial fluid, which contains cations and anions. As illustrated in Fig. 8, macroscale deformation (${\lambda }_{1},{\lambda }_{2}$) results in various changes of the microenvironment with increased tension in the collagen fibers $\Delta T$ and the fixed charge density $\Delta {c}_{{\text{fc}}}$. Further, deformation induces gradients of hydrostatic pressure $\Delta p$, osmotic pressure $\Delta \pi$, and electrical potential $\mathrm{\Delta \Psi }$. Correspondingly, fluid and ionic flows through skin provide additional stimuli for resident cells [46].

Previous studies investigated the response of cells to biophysical stimuli, such as stiffness [47], flow induced shear stress [48, 49], osmotic pressure [50, 51], and hydrostatic pressure [51, 52]. Further, the importance of the time dependence (duration of stimuli) has been demonstrated, for instance regarding the viscoelasticity of the matrix and its role in mechanobiology [53, 54].

The computational analysis of both pregnancy and skin expansion showcased that a change in passive mechanical stretch at the tissue length scales alters a multitude of variables in the biophysical microenvironment of the cells in the dermis. A change in stiffness is predicted by several orders of magnitude, up to values reaching the MPa range. Fibroblasts are known to sense such rigidity changes [55]. For instance, when cultured within matrices with stiffnesses spanning the range reported here, fibroblast proliferation was observed to increase proportionally to matrix stiffness [56]. Changes in osmotic and hydrostatic pressure are in the range of few kPa. While osmotic pressures applied in experimental settings are usually much higher [51, 57], hydrostatic pressures of kPa to MPa were shown to influence cell behavior and, e.g., increase cell proliferation for endothelial cells [58, 59]. Changes in electrical potential are observed in the order of magnitude of few mV. Chondrocytes in cartilage were shown to be sensitive to changes in electrical potential in this range [60, 61]. In human skin, gradients of electrical potential are known to guide cell migration in wound healing [62]. The magnitudes of the gradients usually applied in such experiments are in the order of 100 $\frac{{\text{mV}}}{{\text{mm}}}$. Assuming a cell size of several $\mathrm{\mu m}$ this indeed leads to local differences in electrical potential of few mV. Further, a non-negligible change in volume is predicted by the simulations. Recently, the importance of confinement of cells became more evident [63]. However, the relationship between matrix contraction and dermal cell confinement is difficult to quantify. Altogether, the order of magnitude of several of these cues occurring simultaneously are large enough so that an influence on cell behavior during pregnancy and skin expansion is to be expected.

The differences between mechanome alterations in long-term (pregnancy) and short-term stretching (expansion) may lead to differences in the cellular response. Pregnancy only induces long-term evolutions associated with slow increase of intrabdominal pressure while skin expansion includes subsequent skin stretching events that activate the viscoelastic behavior of skin. As shown in Supplementary Fig. S10, the transient viscoelastic response lasts for several minutes after application of a stretch event. The changes of the mechanome in this early phase may contribute to cell stimulation. In addition, different alterations of gene expression have been observed depending on the duration of the expansion process ranging from weeks to months [38] [64]. In general, the magnitude of change in all biophysical cues is larger in skin expansion than for pregnancy. For instance, for the stiffness the difference in alteration is almost one order of magnitude. For other components, such as hydrostatic pressure and electrical potential, the differences are smaller but may be significant. The temporal evolution of the components of the mechanome represents another important distinctive feature. During pregnancy all cues increase continuously and are likely to exceed the physiological limit only slightly but in a sustained manner. On the other hand, as evident in the results reported in Supporting Information, the fast volume change of the expander (mins) results in a peak of several components of the mechanome, with short-term transient evolutions associated with dissipative processes in the tissue. In fact, the rapid filling of the expander induces non-negligible energy dissipation in the reticular dermis through relaxation of the collagen fibers and fluid flow. Thereby, average flow velocities of few $\frac{\mathrm{\mu m}}{{\text{s}}}$ are expected, which are in the range of values previously shown to induce fibroblast alignment and differentiation [65, 66].

### Model Limitations

While our model is in line with all observations, it relies on several simplifying assumptions. First, it is assumed that the material properties of skin do not change throughout pregnancy or expansion. During short-term application of skin expanders, this might be a valid assumption, as studies reported no significant difference in tissue-scale stiffness between expanded and normal skin [67]. In pregnancy, changes in skin properties due to hormonal fluctuations are possible. Our measurements from the breast and forearm allow to exclude systemic changes, but not local effects in the abdominal skin. Second, the computational results depend on model parameters, which show considerable variability across subjects and body locations. Some of the quantities predicted by the simulation would change to a significant extent with subject specific model calibration, but not the general trends observed in the present analysis. One important uncertainty concerns the exponential behavior of the strain energy function: pregnancy simulations without growth reached an amount of stretch that is well beyond the level of deformation applied for tissue characterization, as skin often ruptures at lower stretch levels. Thus, model extrapolation to such large strains might overestimate the increase in stiffness. On the other hand, the fact that without growth stretches would reach values close or beyond typical rupture stretches in experiments provides another argument for the occurrence of skin growth during pregnancy. Note in this context that alternative model formulations could be considered linking damage of the extracellular matrix with tissue growth and remodeling, as recently proposed in [68]. As a more general limitation, the present study only included a small number of participants, thus affecting the reliability of the reported mean values of skin stiffness during pregnancy and corresponding calculations. Finally, the small cohort does not provide information on the influence of other factors, such as age and body weight.

### Conclusion and Future Work

Increased tension and deformation of human skin during pregnancy was assessed and analyzed both experimentally and computationally. Suction measurements on five subjects at five different body locations allowed to quantify changes in skin stiffness during gestation and after delivery. The computational analysis demonstrated that growth is required during, and resorption after pregnancy to match the experimental results. The analysis of various components of the mechanome showed that they deviate from the reference values, with the largest changes occurring in the last weeks of pregnancy. Skin growth in pregnancy could be rationalized with the same model as the one representing tissue growth during skin expansion. Similar orders of magnitude of alteration were seen for several cues in pregnancy and skin expansion. However, their timeline differs considerably, with time-dependent effects such as fiber relaxation and fluid flow significant only for the case of skin expansion.

The present analysis shows that a stretch-based growth law captures the evolution of tissue dimensions in a phenomenological sense. On the other hand, resident cells are exposed to changes of a variety of chemomechanical variables in their environment which may influence tissue growth. An improved understanding of growth thus requires experimental work to characterize the influence of specific components of the mechanome on the response of cells. In terms of modeling, the present continuum-based approach provides a homogenized representation of all relevant variables in the tissue, while the ECM is highly heterogenous at cell length scale [29, 69]. Hybrid continuum-discrete skin models may offer important insights on the local variability of cell-perceived biophysical cues.

Our study was not intended to provide clinical insights. However, the findings of the present work may be linked with the occurrence of stretch marks in pregnancy, whose causes are not known [70]. Our calculations reveal that, especially in the last trimester of pregnancy, large stresses occur, up to several MPa, which are in the range of experimentally observed failure stresses for skin [71]. This underlines the importance of physiological growth in pregnancy, as it can avoid skin overstretching, possibly reducing the risk of stretch marks. Suction measurements may offer an opportunity to monitor the evolution of skin stiffness and to correlate biomechanical skin condition with the occurrence of stretch marks, thus helping toward their prevention and treatment.

### Supplementary Information

Below is the link to the electronic supplementary material.

Supplementary file1 (DOCX 1201 kb)
