# Supplementary material for: Sustained Physiological Stretch Induces Abdominal Skin Growth in Pregnancy
Source: Ann Biomed Eng. 2024 Feb 29;52(6):1576–90. doi: 10.1007/s10439-024-03472-6 (PMC11081934; doi:10.1007/s10439-024-03472-6)
Supplement: Supplementary file 1 — Supplementary file1 (DOCX 1201 kb) [file 10439_2024_3472_MOESM1_ESM.docx]

# Supporting Information

Sustained physiological stretch induces abdominal skin growth in pregnancy

David Sachs^1,†^, Raphael Jakob^1^, Bettina Thumm^1^, Michael Bajka^2^, Alexander E. Ehret^1,3^, Edoardo Mazza^1,3^

^1^Institute for Mechanical Systems, ETH Zürich, Zurich, Switzerland

^2^Department of Obstetrics and Gynecology, University Hospital of Zurich, Zurich, Switzerland

^3^Empa, Swiss Federal Laboratories for Materials Science and Technology, 8600 Dubendorf, Switzerland

## ^†^ corresponding author

E-mail: sachs@imes.mavt.ethz.ch, mazza@imes.ethz.ch

Keyword: (Skin, Pregnancy, Mechanobiology, Growth, Mechanome, Multiphasic Modeling)

# 1. Mechanical response of skin


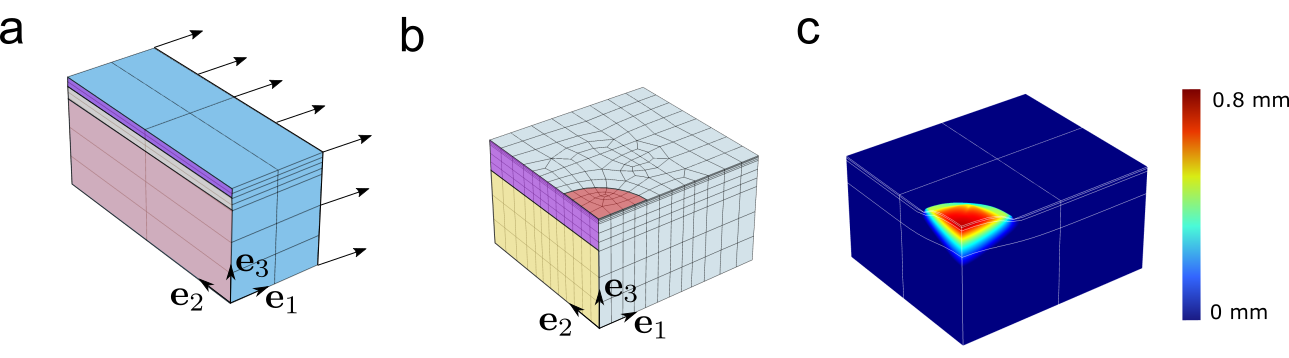


**Figure S1:** Computational analysis of the passive mechanical response of skin. (a) Mesh used for the simulation of uniaxial tensile tests, with indication of epidermis, papillary and reticular dermis. (b) Mesh used for suction simulations. The layers on the side indicate the skin (purple, subdivided in epidermis and papillary and reticular dermis) and the hypodermis (yellow). The area where the negative pressure is applied is indicated in red. (c) Out of plane displacement in a suction simulation.

Figure S1 shows the mesh for uniaxial tension test and suction simulations. Only one quarter of the domain is modelled for uniaxial tension. Symmetry conditions for displacement and fluid flow are imposed on faces with outward normal $-\boldsymbol{e}_{1}$ and$-\boldsymbol{e}_{2}$. No outflow is allowed through the top surface (epidermis), as well as in direction of loading (positive outward normal $\boldsymbol{e}_{1})$. On all remaining boundaries a free deformation boundary for the solid and a pressure boundary condition ${(\mu}_{f}=0)$for the fluid is applied. The displacement is applied as indicated with the arrows in Figure S1(a). The three layers of skin (epidermis, papillary dermis and reticular dermis) are highlighted. Figure S1(b, c) provide information about the suction simulation. Again, one quarter of the domain is simulated with symmetry conditions applied for displacement and fluid chemical potential on faces with outward normal $-\boldsymbol{e}_{1}$ and$-\boldsymbol{e}_{2}$. A layer of adipose tissue is considered for the simulation. Preliminary calculations have shown that the boundary conditions imposed at the bottom surface to represent a tight or loose connection with the underlying muscles have only marginal influence on the simulation results. A negative pressure is applied at the upper surface as indicated by the red area in Figure S1(b). No flow is allowed through the top surface, while on the remaining faces a pressure boundary condition $(\mu_{f}=0)$ is applied for the fluid chemical potential. A ring of 2 mm width around the red area is fixed to simulate the contact of the device with the skin. A deformed state is shown in Figure S1(c).

Data from uniaxial and biaxial (suction) experiments were used to determine model parameters for each layer, see Table S1. The values are based on our previous work [1]. The parameter $q$ was adapted for the three upper layers to reflect the average suction response observed in the present experiments. *In order to further verify the predictive capabilities of the model, we performed suction experiments on the volar forearm for three different configurations, viz. reference, extended arm and stretched skin, see Figure S2. Stretch was applied to skin as described in previous experiments [4]. Thereby we quantified the level of skin stretch with respect to the reference configuration using digital image correlation. We then imposed the corresponding deformation state in the simulations. Stretching induced a significant increase in closing pressure which could be reproduced to a good extent by our model, thus confirming its validity.*


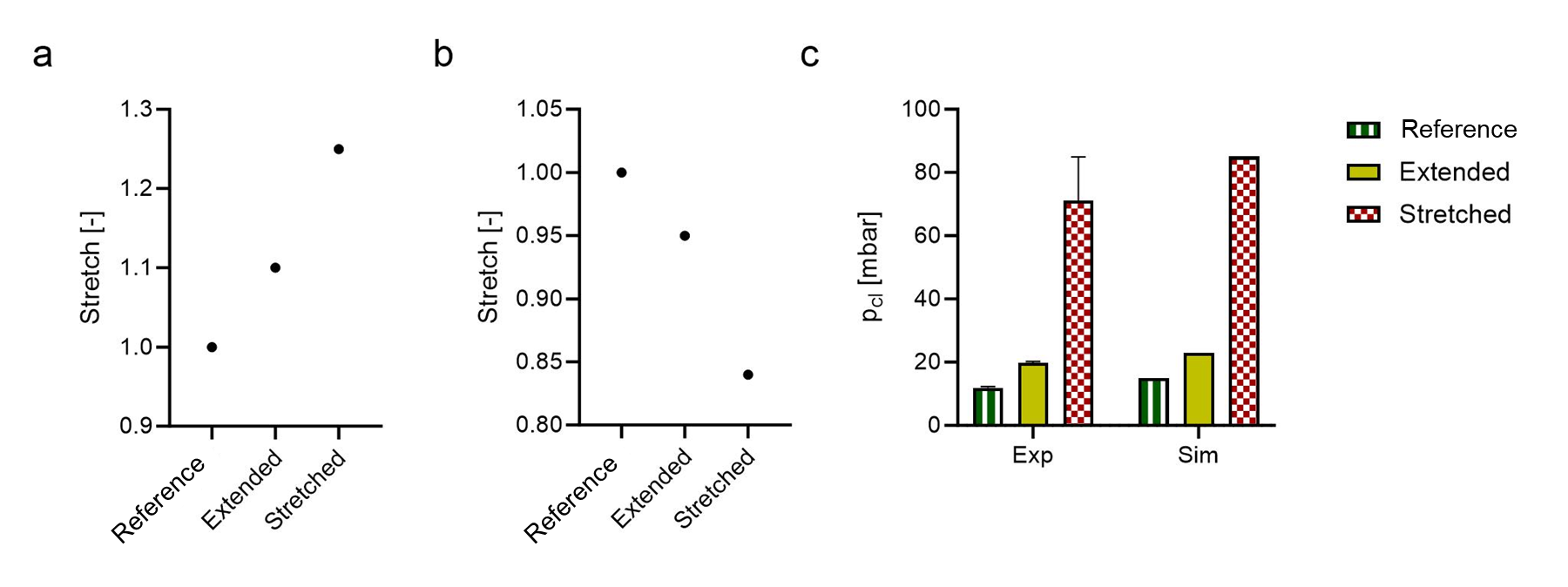


**Figure S2:** Validation of the model on the volar forearm. (a) Stretches measured in direction of the main axis of the volar forearm and (b) perpendicular to it. (c) Closing pressure measured with the Nimble for all three configurations (Exp) and corresponding values of the simulation (Sim).

Additionally, we performed simulations of creep and relaxation experiments of the skin to examine the influence of the time-dependent part of the model, shown in Figure S3. To evaluate the response to creep and relaxation in skin expansion and pregnancy we considered two loading rates: force (0.5 N) and elongation (15%) were applied within 1 min to simulate an expansion step and within 1 day to simulate the slow loading rate in pregnancy. The results demonstrate that time dependent response occurs after faster loading (skin expansion) while neither creep nor relaxation follow the slow loading of pregnancy.**
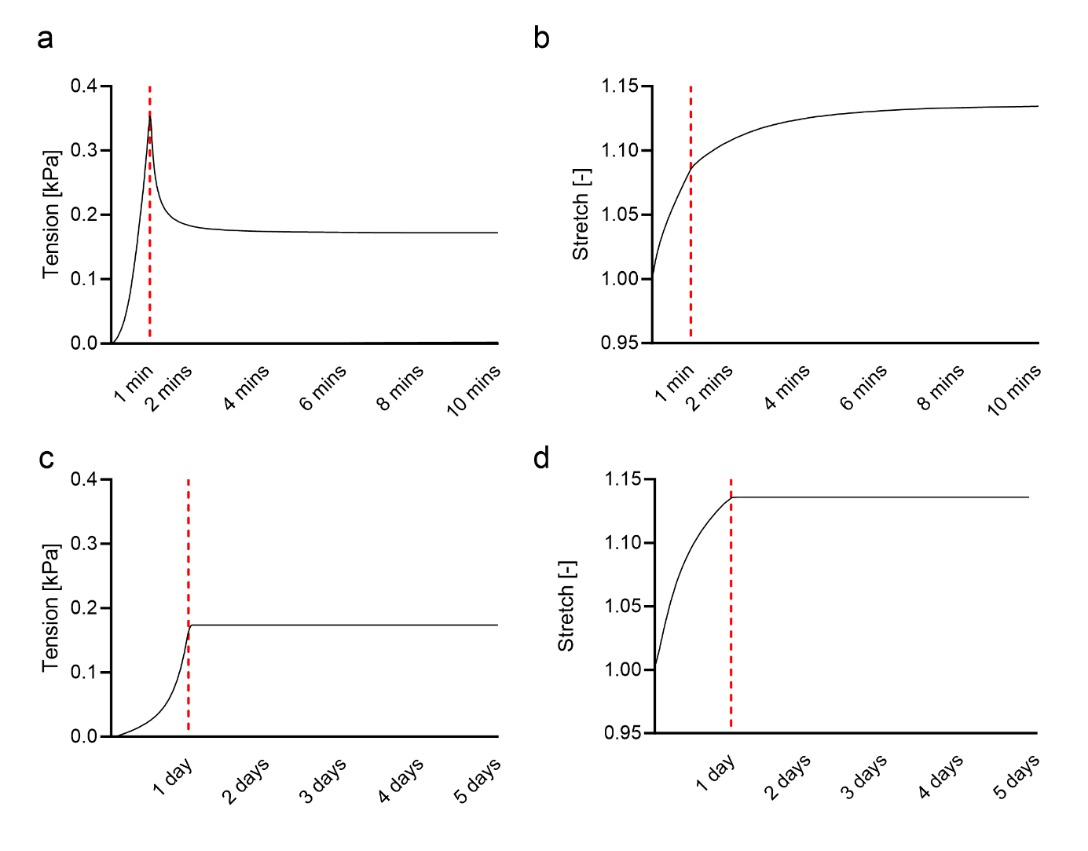
**

**Figure S3:** (a) Relaxation and (b) creep simulation with loading applied within 1 min, corresponding to load rates observed in skin expansion. Both relaxation and creep is observed for several minutes after full loading is applied (red line). (c) Relaxation and (d) creep simulation with loading applied within 1 day corresponding to load rates observed in pregnancy. Neither relaxation nor creep occurs after the loading phase has been completed.

**Table S1.** Material Parameters of the biphasic model for epidermis, papillary and reticular dermis and adipose tissue

|  | Epidermis | Papillary dermis | Reticular dermis | Adipose tissue |
| --- | --- | --- | --- | --- |
| $\mu_{0}\left( \mathrm{kPa} \right)$ | 5.08 | 2.068^)^ | 6.68 | 0.2 |
| $q$ | 1.2 | 1.2 | 1.2 | 1.4 |
| $m_{1}$ | 0.2 | 0.1 | 0.23 | 0.05 |
| $m_{2}$ | 2 | 1 | 1 | 0.1 |
| $m_{\mathrm{fe}}$ | 25 | 110 | 240 | 50 |
| $m_{\mathrm{fd}}$ | 120 | 250 | 1150 | 50 |
| $m_{4e}$ | 2.5 | 1.45 | 1.34 | 1.4 |
| $m_{4d}$ | 1.34 | 1.34 | 1.34 | 1.4 |
| $\theta\left( ^{\circ} \right)$ | 30 | 10 | 7 | 10 |
| $k_{fd}\left( mm^{2}N^{-1}s^{-1} \right)$ | 0.0143 | 0.143 | 0.0143 | 5.43 |
| $k_{0}\left( mm^{4}N^{-1}s^{-1} \right)$ | 0.005 | 0.05 | 5 | 15 |
| $\kappa$ | 2 | 2 | 2 | 2 |
| $\beta_{0}\left( \mathrm{kPa} \right)$ | 0.124693 | 1.9170193 | 2.49 | 0.0065316 |
| $\beta_{1}$ | 2.0 | 2.0 | 2.0 | 2.0 |
| $\varphi_{s}^{\mathrm{ref}}$ | 0.3 | 0.3 | 0.3 | 0.3 |

# 2. Experimental data

In the following, the full set of measurements on pregnant subjects is reported. The results of suction measurements are shown for the abdomen (Figure S4), the breast (Figure S5) and the volar forearm (Figure S6).


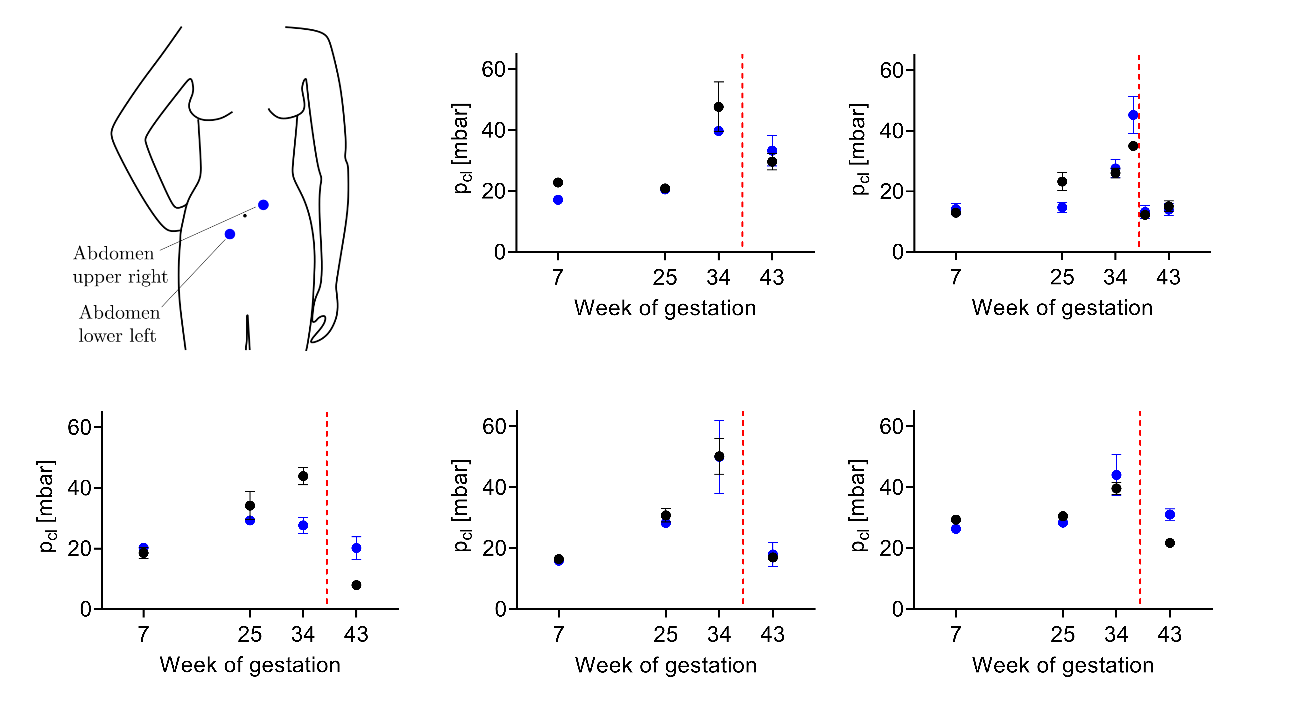


**Figure S4.** Results of suction measurements on the abdomen for each study participant.


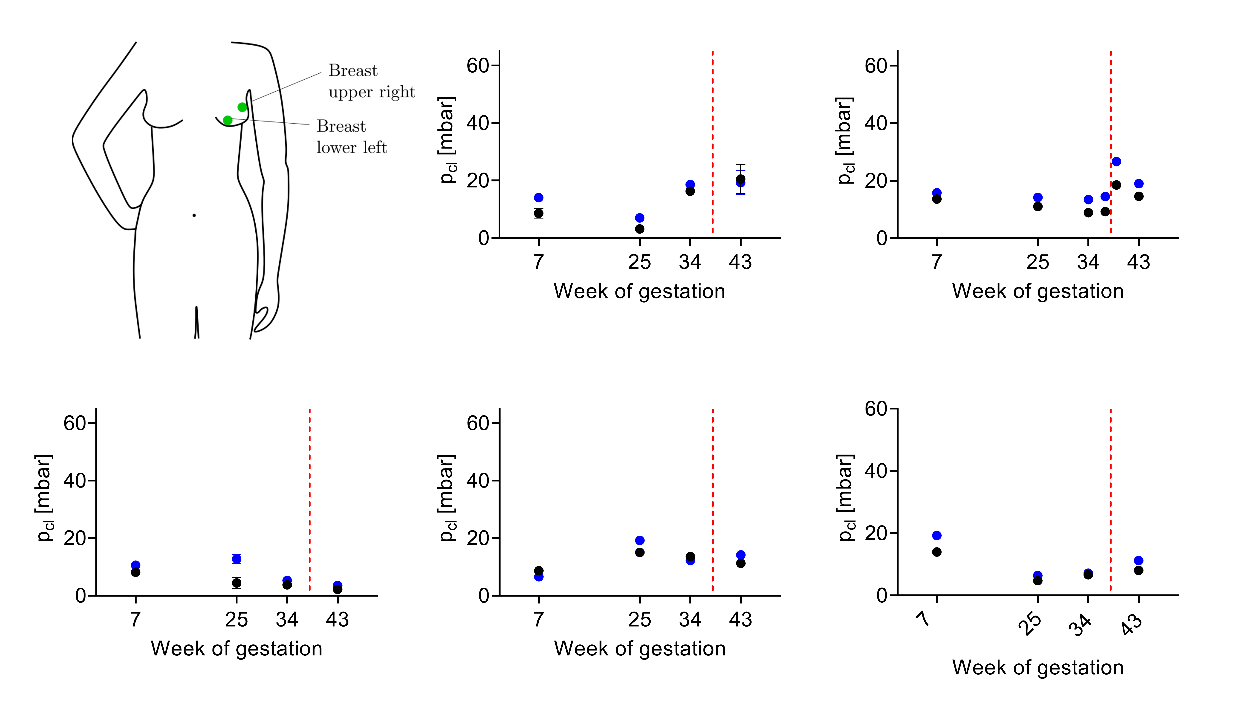


**Figure S5.** Results of suction measurements on the breast for each study participant.


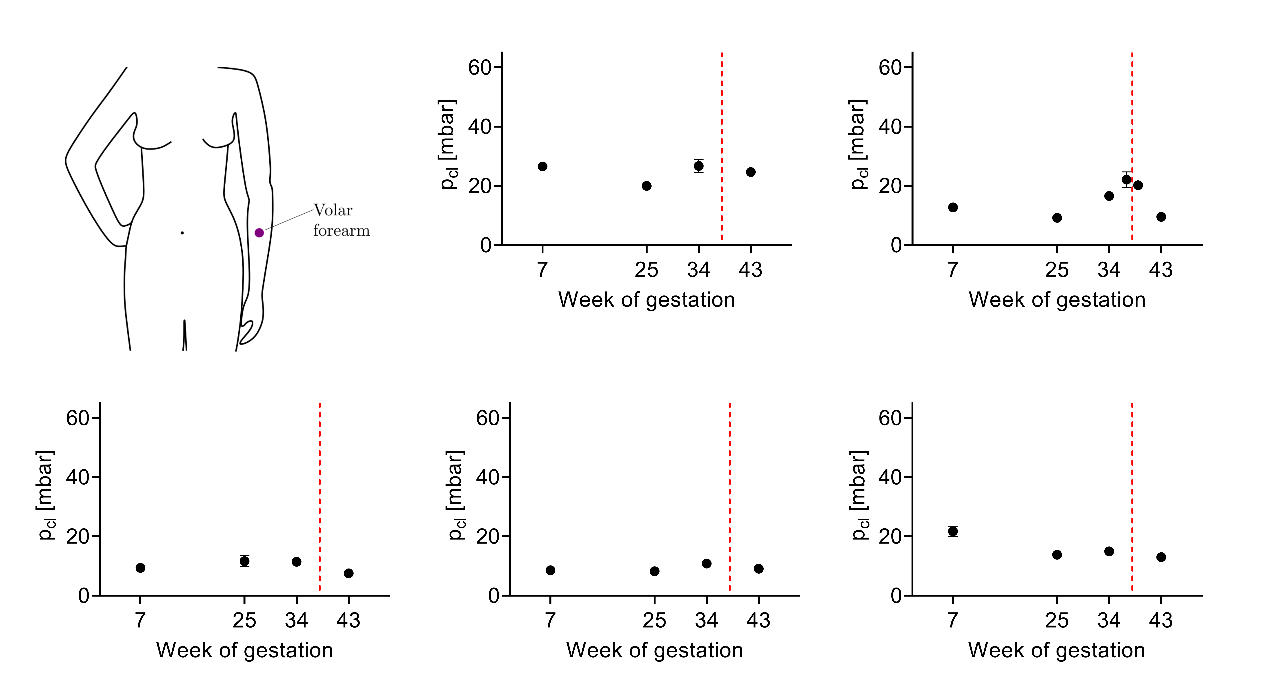


**Figure S6.** Results of suction measurements on the volar forearm for each study participant.

## 3. Derivation of additional state variables of the mechanome

## 3.1 Solid and fluid dissipation

The biphasic material has two sources of dissipation: the one due to fluid flow and the dissipative fiber deformation. The fluid dissipation rate per unit volume $\left[ \frac{J}{m^{3}s} \right]$ results from Darcy’s law with [2]

$D_{F}=\varphi_{f}^{2} \boldsymbol{k}^{-1}\boldsymbol{j}_{f} \cdot\boldsymbol{j}_{f}.$ (S1)

Therein $\varphi_{f}$ corresponds to the current fluid volume fraction, $\boldsymbol{k}$ is the spatial permeability tensor and $\boldsymbol{j}_{f}$ the interstitial fluid velocity.

The solid dissipation rate per unit volume $\left[ \frac{J}{m^{3}s} \right]$ stemming from the dissipative fibers is

$D_{S}=k_{\mathrm{fd}}\frac{1}{N}\sum_{i=1}^{N} \left[ \frac{\varphi_{s}}{J_{\mathrm{pm}}}\mu_{0}\exp\left( qg \right)m_{\mathrm{fd}}\left\langle\lambda_{\mathrm{fd}}^{i}-1 \right\rangle^{2m_{4}-1}\lambda_{\mathrm{fd}}^{i} \right]^{2}$ (S2)

where $J_{\mathrm{pm}}$ is the determinant of $\boldsymbol{F}_{\mathrm{pm}}$, $\lambda_{\mathrm{fd}}^{i}$ is the stretch of the fibers, which is calculated according to the evolution equations provided in [3], [4], $\varphi_{s}$ is the volume fraction of the solid compartment, and *N* is the number of dissipative fiber families.

## 3.2 Concentration of the ions and electrical potential

The concentrations of the anions and cations as well as the electrical potential inside the tissue can be derived based on Donnan’s equilibrium [5], [6]. Assuming equilibrium with an external bath with ion concentration $c_{m}$ , the ion concentrations in the tissue follow as [6]

$c_{+}\left( J_{\mathrm{pm}} \right)=\sqrt{c_{m}^{2}+\left( \frac{c_{\mathrm{fc}}\left( J_{\mathrm{pm}} \right)}{2} \right)^{2}}+\left( \frac{c_{\mathrm{fc}}\left( J_{\mathrm{pm}} \right)}{2} \right),$ (S3)

$c_{-}\left( J_{\mathrm{pm}} \right)=\sqrt{c_{m}^{2}+\left( \frac{c_{\mathrm{fc}}\left( J_{\mathrm{pm}} \right)}{2} \right)^{2}}-\left( \frac{c_{\mathrm{fc}}\left( J_{\mathrm{pm}} \right)}{2} \right).$ (S4)

The electrical potential inside the tissue can be determined as [7]–[9]

$\Psi\left( J_{\mathrm{pm}} \right)=\frac{RT}{2F}\ln\left( \frac{{\gamma_{-}c}_{-}\left( J_{\mathrm{pm}} \right)}{\gamma_{+}c_{+}\left( J_{\mathrm{pm}} \right)} \right).$ (S5)

Therein, $\gamma_{+}=\gamma_{-}=1$ are the activity coefficients of anions and cations, $F$ is Faraday's constant, $R$ is the ideal gas constant, and $T$ the temperature. All quantities are connected to the current deformation of the tissue through the volume dependence of the fixed charge density

$c_{\mathrm{fc}}\left( J_{\mathrm{pm}} \right)=\frac{1-\varphi_{s}^{\mathrm{ref}}}{J_{\mathrm{pm}}-\varphi_{s}^{\mathrm{ref}}}c_{\mathrm{fc}}^{\mathrm{ref}}.$ (S6)

Therein, $\varphi_{s}^{\mathrm{ref}}=0.3$ is the initial volume fraction of the solid and $c_{\mathrm{fc}}^{\mathrm{ref}}=0.025 M$ is the fixed charge density in the reference configuration.

The predicted evolution of anion and cation concentrations are shown in Figure S7. Due to skin volume decrease in pregnancy, anion concentration decreases and cation concentration increases, especially in the last trimester. The maximum deviation from the initial value is 0.0025 M for both ions. In skin expansion, the temporal behavior of anions and cations follows the expansion cycles. The maximum changes of ion concentrations are about 0.004 M. The first few minutes after inflation reveal that also for ions there is a peak of concentration at the end of the inflation, which relaxes within the first minutes after inflation.


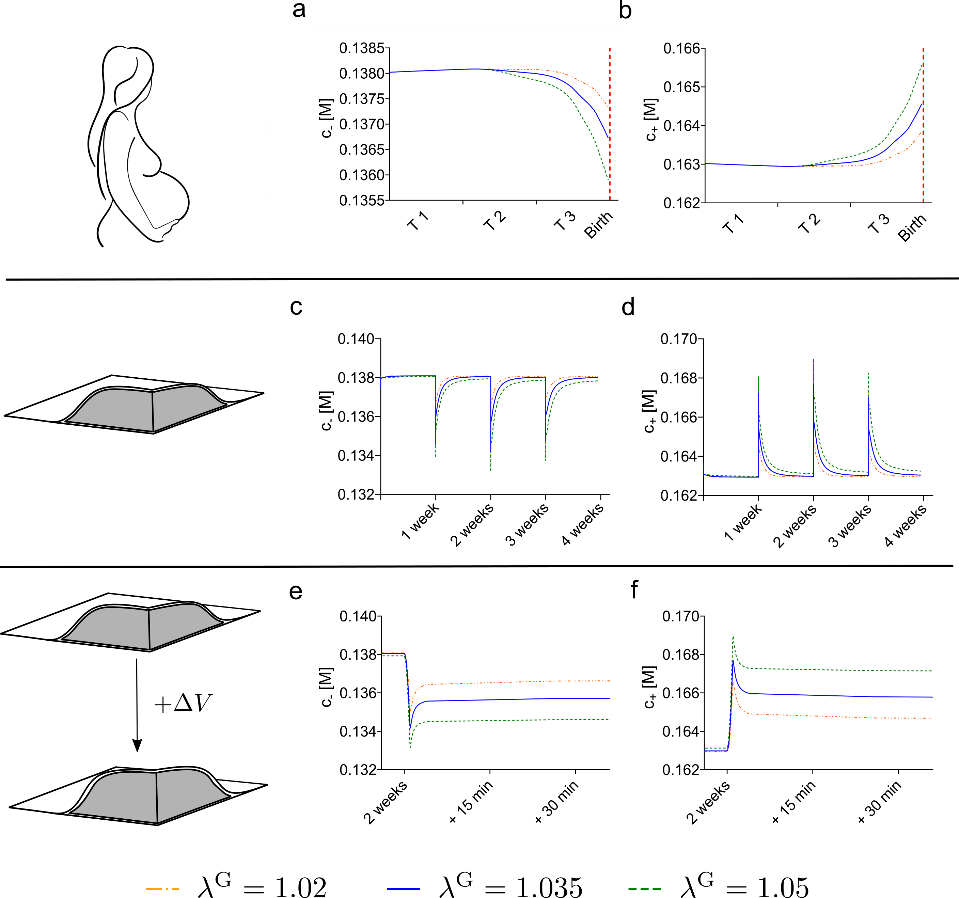


**Figure S7**: Anion and cation concentrations vary in pregnancy and skin expansion for different values of $\lambda^{G}$ . (a) Concentration of anions and (b) cations during pregnancy, and (c) concentration of anions and (d) cations during skin expansion. Changes of concentration in the first 30 minutes after the expansion step are shown in (e) for anions and (f) for cations.

## 3.3 Perfusion Velocity

Cells residing in human skin are supplied with nutrients by a constant interstitial fluid flow. Fluid thereby leaks from the vascular system into the interstitial space and is reabsorbed by the lymphatic capillaries [10], [11]. The perfusion velocity calculates as

$v_{p}=\frac{k\left( J_{\mathrm{pm}} \right)\left( p_{b}-p_{l} \right)}{l_{0}}.$ (S7)

Therein $p_{b}$ is the pressure difference between the chemical potential of the interstitial space and the capillaries, and $p_{l}$ the pressure difference between the chemical potential of the interstitial space and the lymphatic capillaries. The characteristic length between venous and lymphatic capillaries is denoted with $l_{0}$. The spatial deformation dependent permeability is given by

$k=k_{0}\frac{\left( J_{\mathrm{pm}}-\varphi_{s}^{\mathrm{ref}} \right)}{\left( 1-\varphi_{s}^{\mathrm{ref}} \right)}.$ (S8)

The following values are used for the parameters: $p_{b}=0.25 \mathrm{kPa}$, $p_{l}=0.25 \mathrm{kPa}$*,* $l_{0}=100 \mu m$*,* $k_{0}=0.5\frac{mm^{4}}{\mathrm{Ns}}$[12]–[14]

# 4 Finite element simulations of pregnancy and skin expansion

## 4.1 Pregnancy simulations

The finite element model is implemented in COMSOL Multiphysics™ (COMSOL Multiphysics 6.0™, COMSOL AB, Stockholm Sweden). The simulation is separated into several steps as shown in Figure S8(a). In a first step the tissue swells from the zero-energy state to the swollen state, due to a nonzero osmotic pressure. The swelling results in the stretch $\lambda_{\mathrm{ip}}$ in both in-plane directions and $\lambda_{\mathrm{op}}$ in the out-of-plane direction. As a next step, an in vivo prestretch $\lambda_{n}$ is applied in both in-plane directions, leading to in-plane elongation and out-of-plane contraction. In a third step, pregnancy is simulated by applying displacement boundary conditions, effectively increasing the imposed in-plane stretch. After delivery, the same displacement boundary conditions are applied as at the beginning of pregnancy. Suction simulations are performed for the gestational weeks 7, 25, 34 and 6 weeks post-partum.

A cuboid of $10\times10\times3 mm^{3}$ is modeled as shown in Figure S8(b), representing ¼ of the full domain. The cube is divided vertically into four layers. The uppermost 100 $\mu m$ represent the epidermis, followed by a 200 $\mu m$ thick papillary dermis, a 1.7 mm thick reticular dermis and finally 1 mm of adipose tissue. Symmetry conditions are applied for both fluid and solid components on boundaries with outward facing normal $-\boldsymbol{e}_{1}$ and$-\boldsymbol{e}_{2}$. Displacement conditions are applied for the solid domain and free flow conditions are applied for the fluid domain on boundaries with outward normal $\boldsymbol{e}_{1}$ and$\boldsymbol{e}_{2}$. The top side, representing the epidermis, is free to move, however no flow is allowed through this side. The bottom side representing the adipose tissue is free to move and fluid is free to flow. Data are evaluated at the center of the specimen on the red line. The data for each layer is evaluated at the center of each layer. For the suction simulation the domain is re-meshed as shown in Figure S8(c). A suction pressure is applied on a quarter circle with a radius of 3 mm. The other parts of the top surface are fixed at a specified displacement resulting from the pregnancy simulation. All other boundary conditions remain the same as for the pregnancy simulation. The apex elevation of skin due to suction is evaluated at the center of the model represented by the red dot.


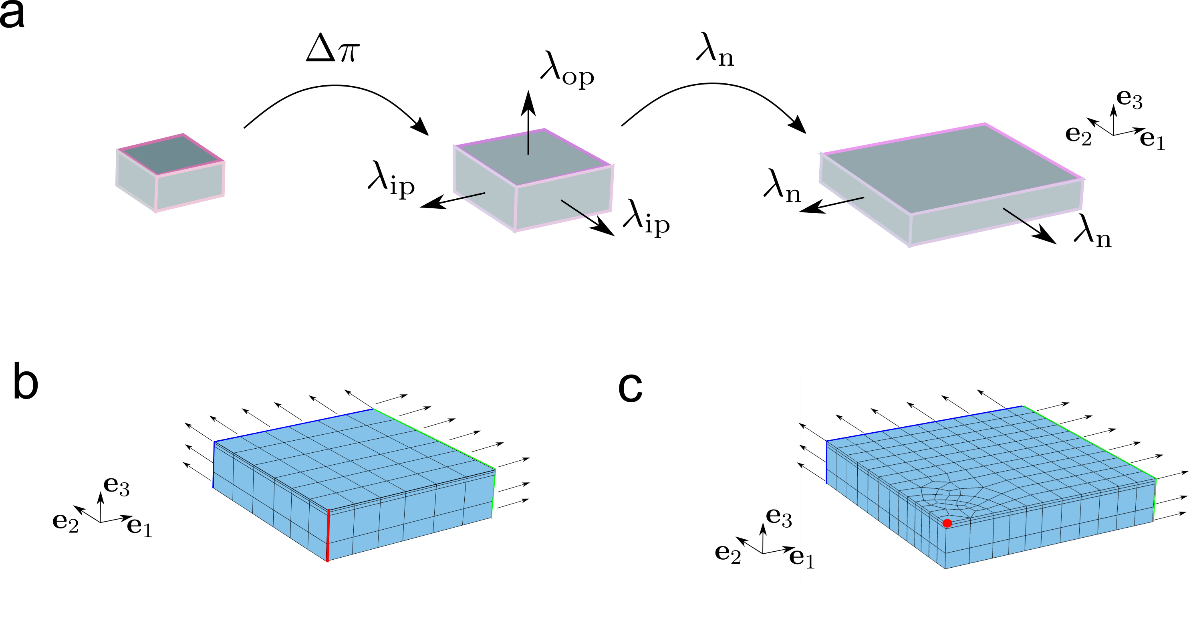


**Figure S8*.*** (a) Two initial steps are performed to reach the in-vivo reference configuration. The material first swells from the zero-energy configuration to the swollen reference configuration, due to a non-zero osmotic pressure. Afterwards an in-vivo stretch $\lambda_{n}$ is applied equibiaxially to reach the in-vivo reference configuration. (b) Mesh and boundary conditions for the pregnancy simulation. Deformation and chemomechanical quantities were evaluated in the center of each layer along the red line. (c) Mesh and boundary conditions for the suction simulation of the pregnancy study. The apex elevation is evaluated at the red dot.

## 4.2 Skin expander simulations


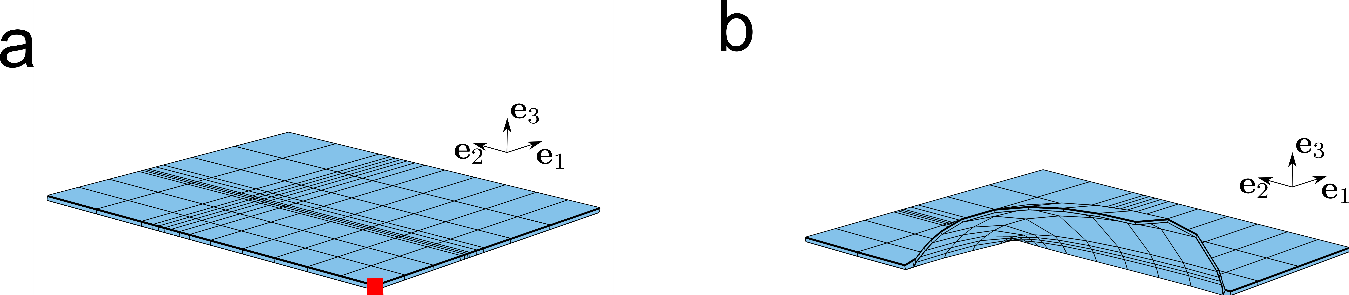


**Figure S9.** Finite element model for skin expansion in the (a) initial state and (b) expanded state. The area of the expander is visible as the mesh is refined at the edges of the expander. The red dot in (a) denotes the point at which stretches and components of the mechanome were evaluated.

The finite element model implemented for skin expansion simulations is shown in Figure S9. Only epidermis, papillary and reticular dermis are considered. Like the simulation of pregnancy, the in vivo configuration is obtained by first applying an osmotic swelling to the system followed by an equibiaxial prestretch. The thickness of each layer is the same as for the pregnancy simulation. Symmetry conditions are applied on both lateral sides facing the reader to model only $\frac{1}{4}$ of the full domain. The modelled total domain is $150\times150\times2 mm^{3}$. An inner rectangle of $60\times100 mm^{2}$ represents the initial area of the empty expander. Both back lateral sides are fixed, and fluid is free to flow through them. At the bottom fluid is free to flow. The displacement for all bottom surfaces outside the expander is prescribed using a spring foundation. The inflation steps of the skin expander are simulated by prescribing the volume of the whole region below the expander using COMSOL’s Global Equation tool. The volume is thereby directly prescribed under the assumption that the expander is filled with an incompressible fluid. This results in the introduction of a Lagrange parameter representing the pressure acting on the skin from the expander which is solved for. The point for data acquisition is situated in the middle of each layer at the center of the expander.

# 5. The mechanome of skin immediately after the expansion step

To better illustrate the magnitude and duration of the transient effects immediately after skin expansion Figure S10 shows the stretches and the biomechanical cues for the first 30 mins after the $3^{rd}$ inflation step, meaning 2 weeks after the start of the procedure. The in-plane stretches in (a) and (b) show that the inflation step results in increased passive mechanical deformation, while no significant growth occurs in that short period of time. The out-of-plane stretch (c) shows an initial contraction to 0.67 which quickly recovers to 0.7 and then stays constant. This rapid deformation is linked with dissipative effects of the fibers (f) and fluid flow (h). Both contribute to a relaxation of the biomechanical cues in the first few minutes. The inflation step induces flow velocities of up to 2 $\frac{\mu m}{s}$ for several minutes within the reticular dermis. The dissipation has a large impact on the stiffnesses in both principal directions as they reduce within few minutes from 5 and 4 MPa to 2 and 1.5 MPa, respectively. The transient effects on the other biomechanical cues are less pronounced.


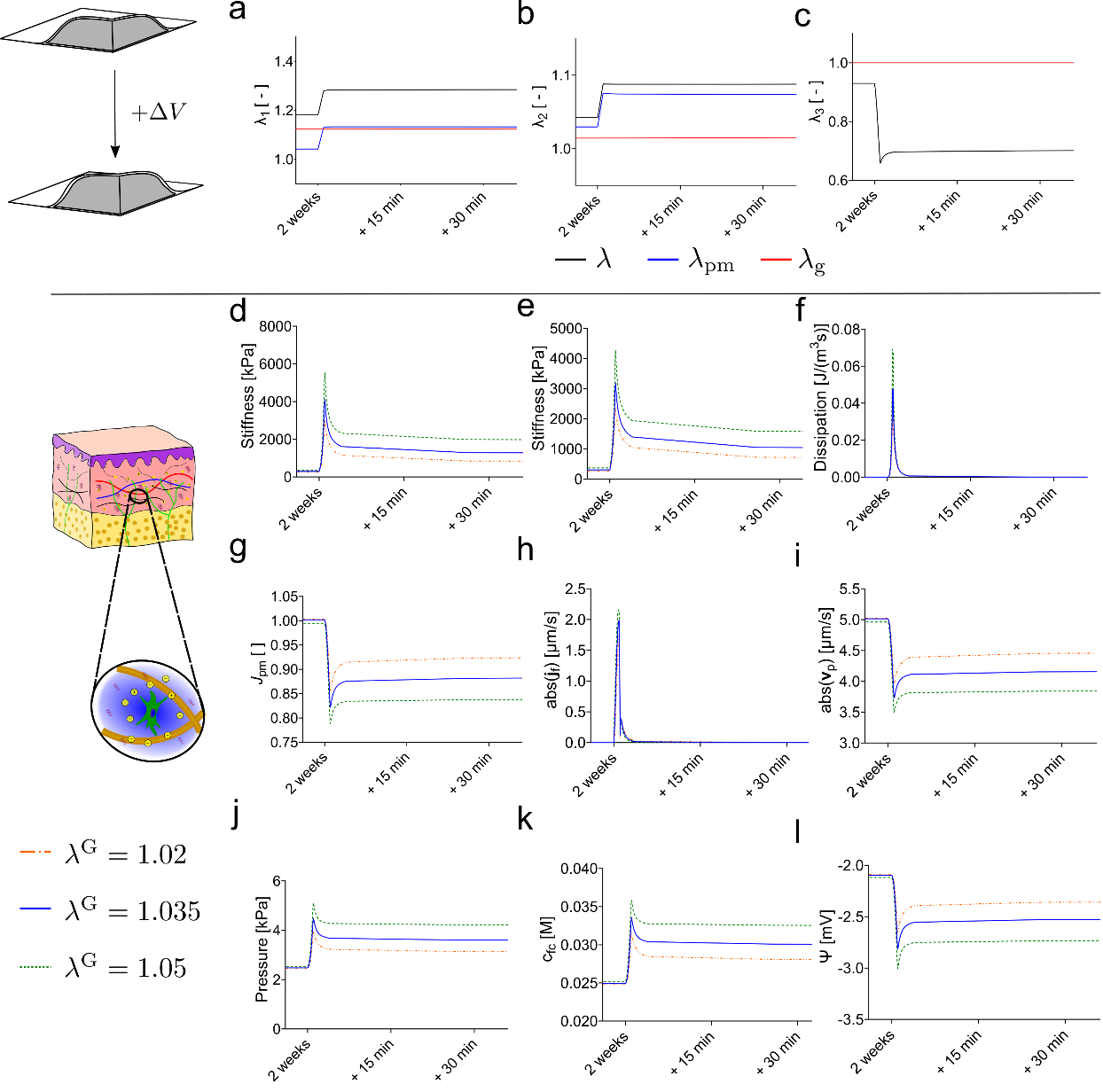


**Figure S10.** Representation of the stretches and components of the mechanome in the first 30 minutes after the expansion. Results are shown for three levels of critical stretch ($\lambda^{G}=1.02,1.035,1.05$). Passive mechanical stretches (blue) and growth stretches (red) are shown for (a) first principal direction, (b) second principal direction (c) out-of-plane direction. (d) Stiffness in first principal direction, (e) stiffness in second principal direction, (f) dissipation of the solid material, (g) volume ratio (h) interstitial fluid velocity, (i) perfusion velocity, (j) osmotic and hydrostatic pressure, (k) fixed charge density, (l) electrical potential.

References

[1] D. Sachs, A. Wahlsten, S. Kozerke, G. Restivo, and E. Mazza, “A biphasic multilayer computational model of human skin,” *Biomech Model Mechanobiol*, vol. 20, no. 3, pp. 969–982, Jun. 2021, doi: 10.1007/s10237-021-01424-w.

[2] A. Wahlsten, “Multiscale mechanics of skin and skin-equivalent materials,” Jan. 2023, doi: 10.3929/ETHZ-B-000589597.

[3] A. Mauri, A. E. Ehret, D. S. A. De Focatiis, and E. Mazza, “A model for the compressible, viscoelastic behavior of human amnion addressing tissue variability through a single parameter,” *Biomech Model Mechanobiol*, vol. 15, no. 4, pp. 1005–1017, Aug. 2016, doi: 10.1007/s10237-015-0739-0.

[4] A. Wahlsten, M. Pensalfini, A. Stracuzzi, G. Restivo, R. Hopf, and E. Mazza, “On the compressibility and poroelasticity of human and murine skin,” *Biomech Model Mechanobiol*, pp. 1–15, Feb. 2019, doi: 10.1007/s10237-019-01129-1.

[5] F. G. Donnan, “Theorie der Membrangleichgewichte und Membranpotentiale bei Vorhandensein von nicht dialysierenden Elektrolyten. Ein Beitrag zur physikalisch-chemischen Physiologie.,” *Zeitschrift für Elektrochemie und angewandte physikalische Chemie*, vol. 17, no. 14, pp. 572–581, Jul. 1911, doi: 10.1002/BBPC.19110171405.

[6] W. Ehlers, N. Karajan, and B. Markert, “An extended biphasic model for charged hydrated tissues with application to the intervertebral disc,” *Biomech Model Mechanobiol*, vol. 8, no. 3, pp. 233–251, Jun. 2009, doi: 10.1007/s10237-008-0129-y.

[7] D. N. Sun, W. Y. Gu, X. E. Guo, W. M. Lai, and V. C. Mow, “A mixed finite element formulation of triphasic mechano-electrochemical theory for charged, hydrated biological soft tissues,” *Int J Numer Methods Eng*, vol. 45, pp. 1375–1402, 1999, doi: 10.1002/(SICI)1097-0207(19990810)45:10.

[8] A. J. H. Frijns, J. M. Huyghe, and J. D. Janssen, “A validation of the quadriphasic mixture theory for intervertebral disc tissue,” *Int J Eng Sci*, vol. 35, no. 15, pp. 1419–1429, Dec. 1997, doi: 10.1016/S0020-7225(97)00047-5.

[9] P. Fievet, “Donnan Potential,” in *Encyclopedia of Membranes*, Berlin, Heidelberg: Springer Berlin Heidelberg, 2016, pp. 577–580. doi: 10.1007/978-3-662-44324-8_1716.

[10] M. Skobe and M. Detmar, “Structure, Function, and Molecular Control of the Skin Lymphatic System,” *Journal of Investigative Dermatology Symposium Proceedings*, vol. 5, no. 1, pp. 14–19, Dec. 2000, doi: 10.1046/J.1087-0024.2000.00001.X.

[11] M. A. Swartz and M. E. Fleury, “Interstitial Flow and Its Effects in Soft Tissues,” *Annu Rev Biomed Eng*, vol. 9, no. 1, pp. 229–256, 2007, doi: 10.1146/annurev.bioeng.9.060906.151850.

[12] A. C. Guyton, H. J. Granger, and A. E. Taylor, “Interstitial fluid pressure,” *Physiol Rev*, vol. 51, no. 3, pp. 527–563, Jul. 1971, doi: 10.1152/physrev.1971.51.3.527.

[13] J. R. Levick, “Flow Through Interstitium and Other Fibrous Matrices,” *Quarterly Journal of Experimental Physiology*, vol. 72, no. 4, pp. 409–437, 1987, doi: 10.1113/expphysiol.1987.sp003085.

[14] M. Spiegel, B. Vesti, A. Shore, U. K. Franzeck, F. Becker, and A. Bollinger, “Pressure of lymphatic capillaries in human skin,” *American Journal of Physiology-Heart and Circulatory Physiology*, vol. 262, no. 4, pp. H1208–H1210, Apr. 1992, doi: 10.1152/ajpheart.1992.262.4.H1208.
